# Synthesis and insecticidal activity of acridone derivatives to *Aedes aegypti* and *Culex quinquefasciatus* larvae and non-target aquatic species

**DOI:** 10.1038/srep39753

**Published:** 2017-01-06

**Authors:** Selvaraj Mohana Roopan, Annadurai Bharathi, Naif Abdullah Al-Dhabi, Mariadhas Valan Arasu, G. Madhumitha

**Affiliations:** 1 Chemistry of Heterocycles & Natural Product Research Laboratory, Department of Chemistry, School of Advanced Sciences, VIT University, Vellore-632014, Tamil Nadu, India; 2 Department of Botany and Microbiology, Addiriyah Chair for Environmental Studies, College of Science, King Saud University, P. O. Box 2455, Riyadh, 11451, Saudi Arabia

## Abstract

A serious Mosquito borne yellow fever is one of the grave diseases which affect the major population. Since there is no specific treatment for yellow fever, there is a necessity to develop an effective agent. The series of acridinone analogues 3 to 5 were synthesized with help of non-conventional microwave heating and confirmed by respective spectral characterization. 5c and 3b showed highest activity to kill 90% of larvae against *A. aegypti* and *C. quinquefasciatus*, respectively. Also the active products were treated to check the mortality of non-target aquatic species. Through the reports of the larvicidal bioassay, compounds 3b against *C. quinquefasciatus* whereas 5c against *A. aegypti* were found to be more active. By keeping this as a platform, further extension of the work can be done to find out a valuable drug for controlling disease vectors.

Mosquito borne diseases represent the remarkable cause for morbidity and mortality in the developing countries^1^. *Aedes aegypti* and *Culex quinquefasciatus* are the two major vectors which cause the dreadful disease such as dengue and yellow fever, lymphatic filariasis respectively. Antiviral therapies for other vector diseases have been evaluated. Whereas the WHO fact sheet (2013) reports that there is no specific treatment for yellow fever, only vaccination is possible which features a range of drawbacks^2^. The key to control these diseases is based on the management of the larval population using the larvicidal agents^3^. However, the usages of chemical substances for the vector control also face an issue of resistance towards those substances^4^. The management of *A. aegypti* during various developmental stages was reported previously^5^,^6^,^7^,^8^. Acridine are the imperative heterocyclic nucleus which is effective against mosquito borne diseases^9^ and also present in the marketed drugs that act as antimalarial agents^10^ which is depicted in Fig. 1. The presence of additional heterocycles may intensify the efficiency of the acridine moiety^11^. The activity of acridine-chalcone hybrid^12^ and the diverse therapeutic efficiency of pyrazole-acridine^13^,^14^ created the interest in construction of the molecules for the yellow fever mosquito vector. The reports for the larvicidal activity of chalcones^15^,^16^, pyrazoles^17^, and nitrogen heterocycles^18^,^19^,^20^ were added connotation to the present work.

In the view of potential biological activities of the above examined heterocycles, we report here the synthesis of dihydroacridinone^20^, **3a–e** and pyrazolo fused acridine isomers, **4a–e** and **5a–e**. Our research group have already been actively involved in the metal free synthesis^19^ of some biologically active molecules and we have them experimented against mosquito larvae. We already published an article stating that dihydroacridinone **1** resulted in better amount of yield and stated high mortality % of larvicidal activity against larval stage of Japanese encephalitis vector, *C. quinquefasciatus*^20^.

However, further work has not been progressed on dihydroacridinone **1**. Our research group, admired by our senior researchers, focused our synthesis towards activated biological product *α, β*-unsaturated carbonyl acridinone moiety. Then, it was reacted with hydrazine hydrate and acetic acid in presence of microwave heating (200 W) for 2 min afforded the fused pyrazolo-acridines. All prepared molecules were subjected to larvicidal activity assay against fourth instar larvae of *C. quinquefasciatus* and *A. aegypti* and also verified against non-target aquatic species like *Cybister tripunctatus asiaticus* (Dytiscidae) and *Notonecta undulata* (Hemiptera: Notonectidae).

## Results and Discussion

**Figure 2:**
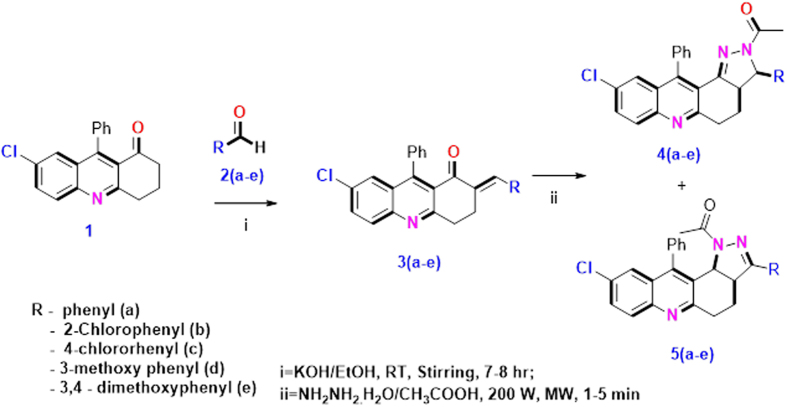
Synthesis of acridinones **4a–e** and **5a–e.**

Synthesis of dihydroacridinone **1** were carried with modified protocol, the mixture of 1,3-hexanedione, 2-amino-5-chlorobenzophenone in the presence of glacial CH_3_COOH and 4 drops of concentrated sulphuric acid was refluxed for 6 h at 150 °C. Furthermore, the intermediate *α, β*-unsaturated ketones, **3a–e** was prepared by condensation of dihydroacridinone **1** in attachment of aromatic aldehyde substation **2a–e** in presence of alcoholic solution. Products **4a–e** and **5a–e** were obtained by further treatment from compounds, **3a–e** with hydrazine monohydrate followed by addition of glacial acetic acid (5 mL) in presence of microwave heating (200 W) for 2 min. The reaction pathway of all the reaction steps are elaborated in Fig. 2 (Table S1). All the synthesized products **3** to **5** were characterized with various spectrometry techniques as mentioned below (^1^H NMR, ^13^C NMR, ESI-MS, Infra-red Spectroscopy, etc.).

IR absorption spectrum of products **3a–e** exhibits –C=O stretching absorption band which appears at 1670–1678 cm^−1^. The peak at 1560 cm^−1^ corresponds to –*C*=*C–* functional group and 3415–3473 cm^−1^ may be due to moisture water peak. These major bands indicate the formation of intermediate products **3a–e** from dihydroacridinone **1**. In the ^1^H NMR spectra, the absence of two protons in acridine ring at *δ* 2.42 ppm, and in ^13^C NMR absence of one aliphatic carbon at 40.71 ppm confirms the formation of products **3a–e**. Furthermore, the target molecule pyrazole isomers were confirmed by the following spectral changes. Compound **4a** has been confirmed by the peak which appear in NMR data i.e., in ^1^H NMR peak at 4.90–4.92, 3.44, 1.75 corresponds to -N-C*H*, -C*H*, -C*H*_*3*_ protons whereas in ^13^C NMR it appears at 66.6, 56.5, and 21.7. Similarly the formation of molecular peak at 452.30 in [M+1] ESI-MS confirms the formation of product **4a**. In FTIR, the band around 1654–1678 cm^−1^ corresponds to the amide carbonyl of products **4a**. It resulted in formation of compound **4a**.

**Figure 3:**
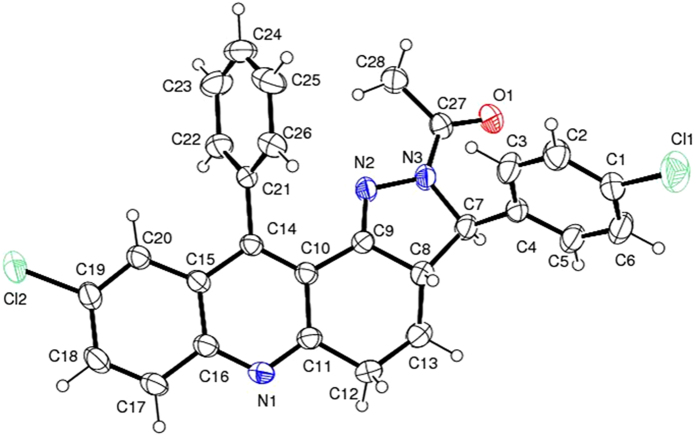
Single crystal structure of compound **4c.**

Furthermore, in ^1^H-NMR doublet peak *δ* 4.9 represents the low polar isomer chiral -N-CH proton, but in the case of high polar product it appears in the down field region at around 5.5 ppm. Other methylene protons present in the acridine core show the presence of two distinct multiplets. From these NMR data, the formation of pyrazole isomers was strongly confirmed. All the compounds have been well characterized by NMR and Mass data. We plan to develop a single crystal for all the possible compounds. Compound 4c has been grown and confirmed by Single crystal XRD as shown in Fig. 3.

The crystal structure displays diverse bond angle & bond length which demonstrate the construction of compound, **4c** in Table S2.

The pyrazole ring formation clearly indicates the following bond length and bond angle of molecular crystal structural data. Two adjacent nitrogen atoms N2–N3 (1.384, 2), nitrogen attached with double bond carbon N2-C9 (1.282, 3) having less bond length compared with nitrogen attached with single bond carbon N3-C7 (1.483, 3), remaining pyrazole ring carbons are C7-C8 (1.554, 3) and C9-C8 (1.504, 3). All aromatic carbon attached with corresponding hydrogens have 0.9300 bond length. The chiral carbon attached hydrogen with bond length of C7-H7 (0.920) and other two aliphatic carbons attached with two hydrogen C12 (HA & HB) and C13 (HA & HB) having the bond length of 0.9700 (Table S3).

**Figure 4:**
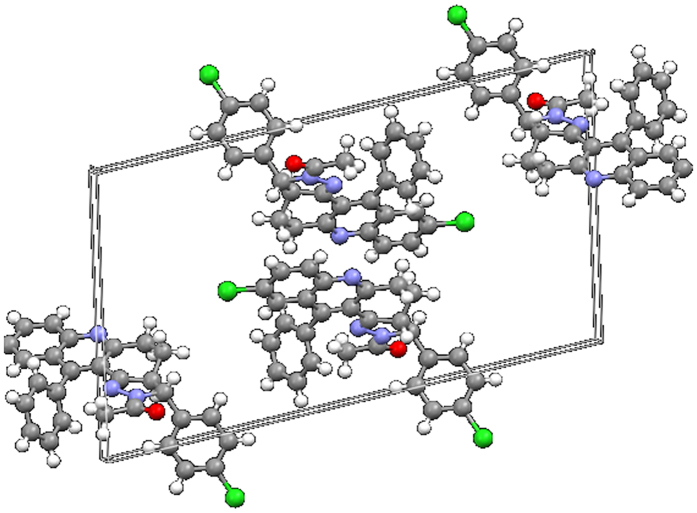
The packing of the molecule in the unit cell, viewed down the a-axis with the hydrogen bond geometry of compound **4c.**

The torsional angles also strongly evidenced the presence of pyrazole ring. The product **4c** was packed by the unit cell with help of molecules which were stated to be geometry of hydrogen bond clearly stated in Fig. 4. The molecular structure of the product **4c** shows that in the pyrazole ring two nitrogen atoms are present in adjacent position. Single crystal structure of product **4c** was shown in Fig. 4. All the above spectral data evidenced the formation of product **4c**.

**1, 3** to **5** were subjected to larvicidal activity assay against each *A. aegypti* and *C. quinquefasciatus* (Table S4). Biological reports stated that several products have significant larvicidal activity against *C. quinquefasciatus* and *A. aegypti*. For example, the significance of mortality observed in **1, 3b**, **3e**, **5c**, **5e**, **4a**, **4c**, and **4d** against *C. quinquefasciatus and A. aegypti* whereas any other compounds did not show positive activity. The values of LC_50_ were 69.94, 43.24, 78.12, and 58.96 ppm against *A. aegypti* and 82.29, 59.12, 92.26, and 76.22 ppm against *C. quinquefasciatus* for **1, 3b**, **3e**, **4a**, and **5c**, respectively. The lowest value of LC_90_ was observed in **5c** (145.70 ppm) against *A. aegypti* and **3b** (186.46 ppm) against *C. quinquefasciatus*. Resulted χ2 numericals stated that those are significant at P < 0.05 level and also confidence limits were identified (LCL and UCL). The mortality of larvae was noted after 24 h exposure.

Larvicidal active compounds such as **1, 3b, 3e, 4a, 4c, 5a, 5b**, and **5c** were tested against two non-target aquatic species to find the toxicity nature of the molecules (Table S5). The results proved that compounds are non-toxic towards these aquatic species.

## Conclusion

In summary, sequences of novel pyrazolo fused acridine isomers **4** and **5** were reported. Overall prepared products were subjected to larvicidal activity against of *C. quinquefasciatus* and *A. aegypti* which resulted in moderate activity. The bioassay result clearly suggested that the maximum activity was exhibited by the product **3b** and **5c** against *C. quinquefasciatus* and *A. aegypti*. Results state that the compounds; **3b** and **5c** proved to be effective agents for discovering a solution for controlling vector mosquitoes.

## Experimental section

### General experimental procedure

#### Synthesis of dihydroacridine [1]

Dihydroacridinone **1** was synthesized as per our earlier report and the samples are authenticated with reported compounds [20].

#### Synthesis of α,β-unsaturated ketones [3a–e]

1 mmol of a 7-chloro-3,4-dihydro-9-phenyl acridin-1(*2 H*)-one **1** was mixed against 1 mmol of aldehydes in aromatic form **2a–e** substituted in 10% alcoholic potassium hydroxide (1 g in 10 mL ethanol). Followed mixture reaction was mixed using stirrer at RT for 7–8 h. Then it was placed in ice cubes and hydrolyzed using HCl. The resultant precipitations were separated using filtration technique and placed for drying. Crude product was isolated using various solvent extraction techniques by column chromatography.

#### Synthesis of pyrazolo fused acridine [4a–e] & [5a–e]

Prepared products **3a–e** (1 mmol) were mixed with hydrazine hydrate (10 mmol) and glacial acetic acid (10 mL) were mixed in 50 mL and reaction processes were progressed using microwave extractor (200 W) for 2 min. The reaction mixtures were monitored by TLC techniques. Once reaction mixtures were completed, it was cooled to RT, filtered and washed with distilled water and further separated with column chromatography (Table S1).

#### X-ray crystallography

The single crystal of product **4c** was synthesized by evaporation method. Enraf Nonius CAD4-MV31 single crystal X-ray diffractometer was used to record the data. For compound, **4c** crystallographic data were attained.

#### Pharmacological activity

For larvicidal activity assay, we collected C. quinquefasciatus and A. aegypti from standing water, Vellore district (12°56′23″ N, 79° 14′23″ E). The collected samples were authenticated from ZERC, Vellore, Tamil Nadu.

#### Larvicidal bioassay

Stock solution: About 1 mg of products, **4** and **5** was diluted with dechlorinated water (100 mL). Dimethyl sulfoxide has been utilized to make the 2% test solution. World Health Organization 1996 protocol with little modification has been applied for the larvicidal activity. To screen the larvicidal assay, five sets of container with 20 larvae from same species in 249 mL dechlorinated water have been used. About 1.0 mL of products 4 and 5 (3.12 to 500 ppm) was mixed in container. H_2_O and DMSO were kept as negative control. Dead larvae number has been calculated after 24 h of the experiment. The experiment has been replicated thrice and the average has been used to find the mortality (100%) of larvae.

#### Statistical analysis

The probit analysis was used to calculate LC_50_, LC_90_, 95% of lower confidence limit, upper confidence limit, and chi-square value. The result was proved as significant since the value is p < 0.05^21^.

#### Non-target species for bioassay

Two non-target aquatic species such as *N. undulata* (Hemiptera: Notonectidae) and *C. tripunctatus* (Dytiscidae) were used in this study for finding the toxicity nature of products **3** to **5.** The dechlorinated water was kept in the lab at 27 ± 2 °C to maintain the non-target species. The products (50 ppm) were added to the bowl which contains 10 individuals with 1000 mL of dechlorinated tap water. The experiment was carried out in 5 batches with 10 individuals of 2 species. About 1 mL of products **3** to **5** at different concentrations (50, 25, 12.5, 6.25 and 3.12 ppm) were used for this study. The control experiment was carried out with distilled water. The experiment was carried out for 3 times. The survival and swimming response of species under exposure to products **3** to **5** were observed continuously through a week.

## Additional Information

**How to cite this article:** Roopan, S. M. *et al*. Synthesis and insecticidal activity of acridone derivatives to *Aedes aegypti* and *Culex quinquefasciatus* larvae and non-target aquatic species. *Sci. Rep.* **7**, 39753; doi: 10.1038/srep39753 (2017).

**Publisher's note:** Springer Nature remains neutral with regard to jurisdictional claims in published maps and institutional affiliations.

## Supplementary Material

Supplementary Information

## Figures and Tables

**Figure 1:**
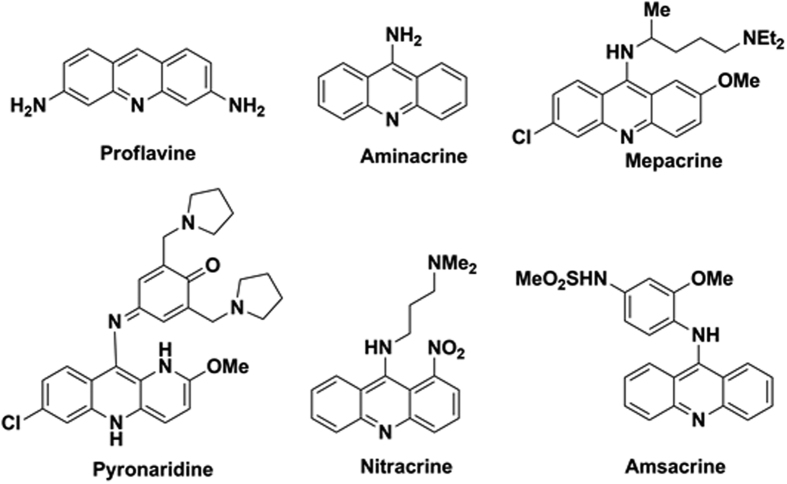
Drugs contain acridine as core moiety.
