# Supplementary material for: Synthesis and insecticidal activity of acridone derivatives to Aedes aegypti and Culex quinquefasciatus larvae and non-target aquatic species
Source: Sci Rep. 2017 Jan 6;7:39753. doi: 10.1038/srep39753 (PMC5216327; doi:10.1038/srep39753)

Synthesis and insecticidal activity of acridone derivatives to *Aedes aegypti* and *Culex quinquefasciatus* larvae and non-target aquatic species

Selvaraj Mohana Roopan [a,*], Annadurai Bharathi [a], Naif Abdullah Al-Dhabi[b], Mariadhas Valan Arasu[b], G. Madhumitha[a]

[a] Chemistry of Heterocycles & Natural Product Research Laboratory, Department of Chemistry, School of Advanced Sciences, VIT University, Vellore-632014, Tamil Nadu, India. [b]Department of Botany and Microbiology, Addiriyah Chair for Environmental Studies, College of Science, King Saud University, P. O. Box 2455, Riyadh, 11451, Saudi Arabia.

* *Corresponding author. E-mail: selvarajmohanaroopan@gmail.com; mohanaroopan.s@vit.ac.in; Tel.: +91-98656-10356; fax: +91-416-224-5544/5766.*

Experimental section

X-ray crystallography

The single crystal of compound (**4c**) was obtained by the slow evaporation of the compound (**4c**) at room temperature from ethyl acetate. The measurements were made on Enraf Nonius CAD4-MV31 single crystal X-ray diffractometer. The diagrams and calculations have been performed using SAINT (APEX II) for frame Integration, SHELXTL for structure solution and refinement software programs. The crystallographic data and structure refinement parameter of compound (**4c**) was explained.

The chiral carbon attached hydrogen with bond length of C7-H7 (0.920) and other two aliphatic carbons attached with two hydrogen C12 (HA & HB) and C13 (HA & HB) having the bond length of 0.9700. All above bond length and angles are summarized in **Table S2 and S3**.

Pharmacological activity

Insect rearing

A. aegypti and *C. quinquefasciatus* larvae were collected from stagnant water area of Melvisharam (12°56′23″ N, 79° 14′23″ E) and identified in Zonal Entomological Research Centre, Vellore (12°55′48″ N, 79°7′48″ E), Tamil Nadu, to start the colony, and larvae were kept in plastic and enamel trays containing tap water. They were maintained and reared in the laboratory as per the reported method.

Dose-response bioassay

From the stock solution, different concentrations ranging from 3.12 to 500 ppm were prepared for larvicidal activity. Based on the preliminary screening results, synthetic compounds, 3a-e, 4a-e, and 5a-e were subjected to dose response bioassay for larvicidal activity against the larvae of *A. aegypti* and C. *quinquefasciatus.* The numbers of dead larvae were counted after 24 h of exposure, and the percentage of mortality was reported from the average of three replicates. However, at the end of 24 h, the selected test samples turned out to be equal in their insect toxic potential.

Statistical analysis

The average larval mortality data were subjected to probit analysis for calculating LC_{50}, LC_{90}, 95 % upper confidence limit and lower confidence limit with chi-square values were calculated using the Probit analysis software. Results with $p<0.05$ were considered to be statistically significant.

Table S1. Synthesis of **4a-e** and **5a-e** from **3a-e**.

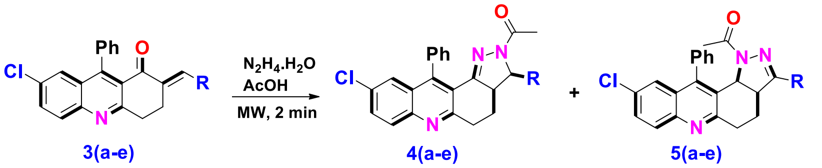

Compounds	Substrate	Product	mp. (°C)	Yield [b]
4a	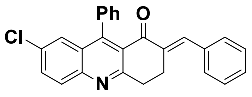	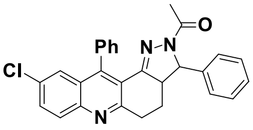	158-160	34
4b	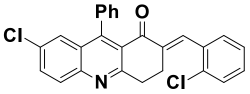	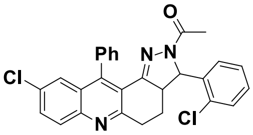	226-228	38
4c	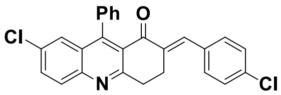	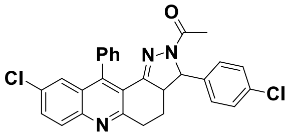	192-194	35
4d	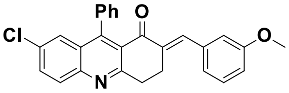	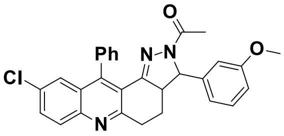	218-220	36
4e	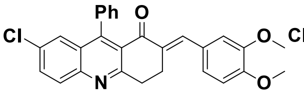	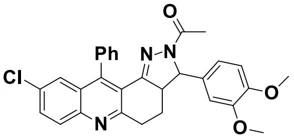	230-232	38
5a	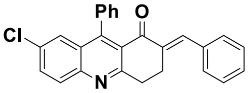	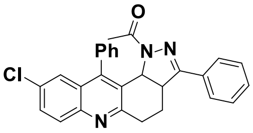	165-167	45
5b	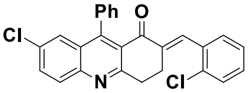	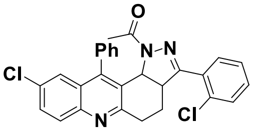	216-218	37
5c	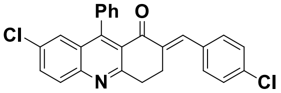	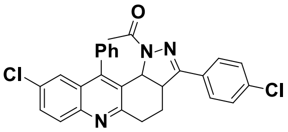	178-180	42
5d	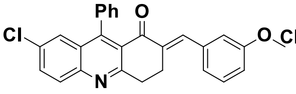	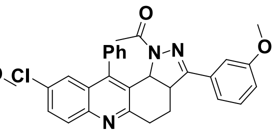	140-142	39
5e	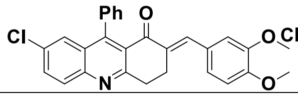	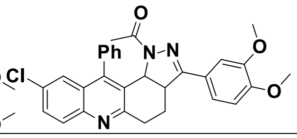	236-238	39

[a] Reactions were carried out with 1.0 equivalence of 3 and 4 in 10 mL glacial acetic acid for 2 min under Microwave heating (200 W). [b] Isolated yields.

Table S2 The crystallographic data and structure refinement parameters of compound, **4c**

Empirical formula	C_{28} H_{21} Cl_2 N_3 O
Formula weight	486.38 gm
Temperature	293(2) K
Wavelength	0.71073 A
Crystal system, space group	Monoclinic, P21/c
Unit cell dimensions	a = 13.299(5) A α = 90.000(5)°
	b = 7.725(5) A β = 101.291(5)°.
	b = 23.464 A γ = 90.000(5)°
Volume	2363.9(18) A^3
Z	4
Calculated density	1.367 Mg/m^3
Absorption coefficient	0.302 mm^{-1}
F(000)	1008
Crystal size	0.30 x 0.20 x 0.20 mm
Theta range for data collection	2.12-25.00°.
Limiting indices	-15 <= h <= 15, -9 < = k <= 9, -20 < = l < = 27
Reflections collected / unique	20352 / 4151 [R(int) = 0.0279]
Completeness to theta = 25.00°	100.0 %
Absorption correction	Semi-empirical from equivalents
Max. and min. transmission	0.9634 and 0.9045
Refinement method	Full-matrix least-squares on F^2
Data / restraints / parameters	4151 / 0 / 315
Goodness-of-fit on F^2	1.027
Final R indices [*I* >2sigma(*I*)]	R_1 = 0.0432, wR_2 = 0.1128
R indices (all data)	R_1 = 0.0558, wR_2 = 0.1230
Largest diff. peak and hole	0.350 and -0.279 e. $A^{\circ -3}$

Table. S3 Selected torsion angles [°] for compound, **4c**

N(3)-C(7)-C(8)-C(9)	4.8(2)
C(13)-C(8)-C(9)-N(2)	-129.7(2)
C(7)-C(8)-C(9)-N(2)	-3.7(3)
C(8)-C(9)-N(2)-N(3)	0.7(3)
O(1)-C(27)-N(3)-N(2)	-170.8(2)
C(28)-C(27)-N(3)-N(2)	11.3(3)
C(9)-N(2)-N(3)-C(27)	178.6(2)
C(9)-N(2)-N(3)-C(7)	3.0(3)

Table S4. Probit analysis of synthetic compounds against fourth instar larvae of *A. aegypti* and *C. quinquefasciatus*

Compounds	Species	LC_{50} ± SE (ppm)	UCL -LCL	LC_{90} ± SE (ppm)	UCL - LCL	χ2 (df=4)
1	i	155.87± 1.45	129.79-169.52	394.12 ± 11.21	486.25-286.26	6.06
	ii	163.50 ± 2.14	131.18-191.25	373.24 ± 24.31	442.23-312.14	7.58
3b*	i	69.94 ± 2.56	79.62-58.10	272.36 ± 38.21	238.03-166.62	12.80
	ii	82.29 ± 2.74	97.22-67.55	186.46 ± 24.14	216.82-165.24	8.62
3e*	i	43.24 ± 4.56	53.62-46.70	186.46 ± 24.14	216.82-165.24	8.62
	ii	59.12 ± 6.04	68.28-47.92	217.21 ± 28.92	252.18-214.24	10.40
5c	i	58.96 ± 2.54	65.30-51.12	145.70 ± 14.30	273.72-127.64	12.04
	ii	76.22 ± 1.74	87.73-46.82	218.34 ± 10.21	324.03-244.12	6.82
5e	i	122.64 ± 6.12	188.28-116.07	464.22 ± 14.30	514.72-347.68	10.14
	ii	106.24 ± 4.24	122.92-90.12	342.34 ± 28.26	434.03-268.66	12.60
4a	i	78.12 ± 2.44	89.63-69.91	184.16 ± 24.92	460.92-324.35	8.20
	ii	92.26 ± 1.06	218.02-164.12	346.22 ± 32.26	562.22-422.02	14.62
4c	i	118.98 ± 2.56	186.89-105.07	345.26 ± 16.34	313.72-247.66	10.24

	ii	142.22 ± 2.74	192.23-96.82	372.34 ± 12.24	386.03-286.12	13.80
4d	i	114.62 ± 4.26	130.22-98.24	368.42 ± 14.08	466.82-165.24	16.02
	ii	139.18 ± 5.06	292.26-120.16	417.28 ± 18.92	612.10-418.24	12.40

Note: Negative Control (H_2O, DMSO) - Nil mortality, Positive Control - Temephos[1] (LC_{50}- 0.042 ppm) Significant at i) *A. aegypti* and ii) *C. quinquefasciatus.* Control - Nil mortality; LC_{50} - Lethal concentration that kills 50 % of the exposed larvae; LC_{90} - Lethal concentration that kills 90 % of the exposed larvae; UCL: Upper confidence Limit; LCL: Lower confidence Limit; $\chi 2$ -Chi-square value, Significant at $P < 0.05$ level.

*- The LC_{90} concentration of 3b for *C. quinquefasciatus* and 3e for *A. aegypti* show similar values after the replication.

Table S5. Toxicity of the synthetic compounds 3b, 3e, 5a, 5b, 4a, 4c at 50 ppm against non-target species, *Cybister tripunctatus asiaticus* and *Notonecta undulata*

Compounds	Percentage of Mortality ± SD	
	Cybister tripunctatus asiaticus	*Notonecta undulata*
1	7.6 ± 1.15	4.50 ± 0.06
3b	2.8 ± 0.16	3.5 ± 1.06
3e	5.8 ± 0.32	8.4 ± 0.62
5a	4.6 ± 0.48	2.4 ± 0.14
5b	7.2 ± 1.14	4.2 ± 0.02
5c	1.6 ± 0.07	1.2 ± 0.02
4a	8.2 ± 1.90	12.4 ± 2.04
4c	2.6 ± 0.05	6.0 ± 0.34

[a] Mean value of three replicates.

References

1. Lima, T.C. *et al.*, Oxime derivatives with larvicidal activity against *Aedes aegypti* L., Parasitol Res **114**, 2883–2891(2015)

The spectral details of compounds **4a-e** and **5a-e** are as follows,

1-(9-chloro-3,11-diphenyl-3,3*a*,4,5-tetrahydro-2*H*-pyrazolo[3,4-*a*]acridin-2yl) ethanone, **4a**

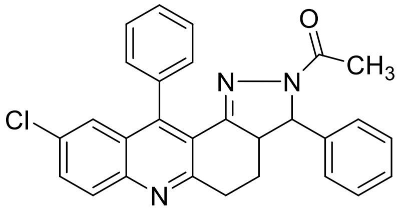

Light yellow powder; M.F: $C_{28}H_{22}Cl_2N_3O$; Yield 34 %; M.P: 158-160 ^{0}C; FT-IR (KBr pellet) $\nu_{max}/(Cm^{-1})$: 1658 (-C=O of tertiary amide); ^{1}H NMR (400 MHz, $CDCl_3$): δ (ppm), 1.75 (s, 3H, $-CH_3$), 2.07-2.15 (m, 1H, $-CH_2$), 2.43-2.46 (t, *J* = 6 Hz, 1H, $-CH_2$) 3.21-3.29 (m, 2H, $-CH_2$), 3.40-3.44 (d, *J* = 17.2 Hz, 1H, prochiral -CH), 4.90-4.92 (d, *J* = 8 Hz, 1H, chiral -N-CH), 7.15-7.17 (d, *J* = 6.8 Hz, 1H), 7.20-7.22 (d, *J* = 6.8 Hz, 3H), 7.31-7.38 (m, 4H), 7.46 (s, 1H), 7.50-7.51 (d, *J* = 6.4 Hz, 1H), 7.54-7.57 (t, *J* = 6.8 Hz, 1H), 7.62-7.64 (d, *J* = 8.8 Hz, 1H), 7.94-7.96 (d, *J* = 8.8 Hz, 1H); ^{13}C NMR (400 MHz, $CDCl_3$) δ (ppm): 21.74, 27.66, 33.98, 56.56, 66.62, 120.93, 125.66, 125.93, 127.78, 2x127.88, 128.04, 128.30, 128.36, 128.94, 2x129.08, 130.02, 130.42, 131.46, 132.60, 136.85, 141.47, 146.19, 146.47, 152.46, 159.04, 170.09; Exact Mass: 451.15; Found ESI-MS m/z: 452.30 [M+1].

1-(9-chloro-3-(2-chlorophenyl)-11-phenyl-3,3*a*,4,5-tetrahydro-2*H*-pyrazolo[3,4-*a*]acridin-2-yl)ethanone, 4b

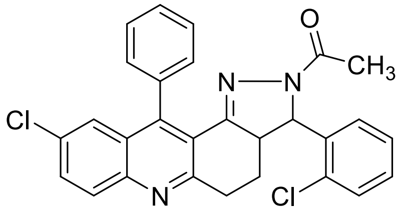

White powder; M.F: $C_{28}H_{21}Cl_2N_3O$; Yield 38 %; M.P: 226-228 ^{0}C; FT-IR (KBr pellet) $\nu_{max}/(Cm^{-1})$: 1678 (-C=O of tertiary amide); ^{1}H NMR (400 MHz, $CDCl_3$) δ (ppm), 0.85-0.89 (m, 1H, $-CH_2$), 1.19-1.30 (m, 1H, $-CH_2$), 1.70 (s, 3H, -CH_3), 1.97-2.02 (m, 1H, $-CH_2$), 3.19-3.34 (m, 1H, $-CH_2$), 3.78-3.86 (m, 1H, prochiral -CH), 5.95-5.98 (d, *J* = 11.6 Hz, 1H, chiral -N-CH), 6.75-6.77 (d, *J* = 7.2 Hz, 1H), 7.15-7.21 (m, 3H), 7.35-7.40 (m, 3H), 7.47-7.55 (m, 2H), 7.58-7.64 (m, 2H), 7.93-7.95 (d, *J* = 8.8 Hz, 1H); ^{13}C NMR (400 MHz, $CDCl_3$) δ: 21.25, 23.26, 33.81, 49.07, 59.62, 120.39, 125.82, 126.66, 127.12,

127.88, 128.11, 128.49, 128.87, 128.92, 129.25, 129.85, 130.19, 131.42, 131.41, 132.16, 132.41, 134.33, 137.18, 146.01, 146.83, 153.00, 158.95, 168.83; Exact Mass: 485.11; Found ESI-MS m/z: 486.27 [M+1].

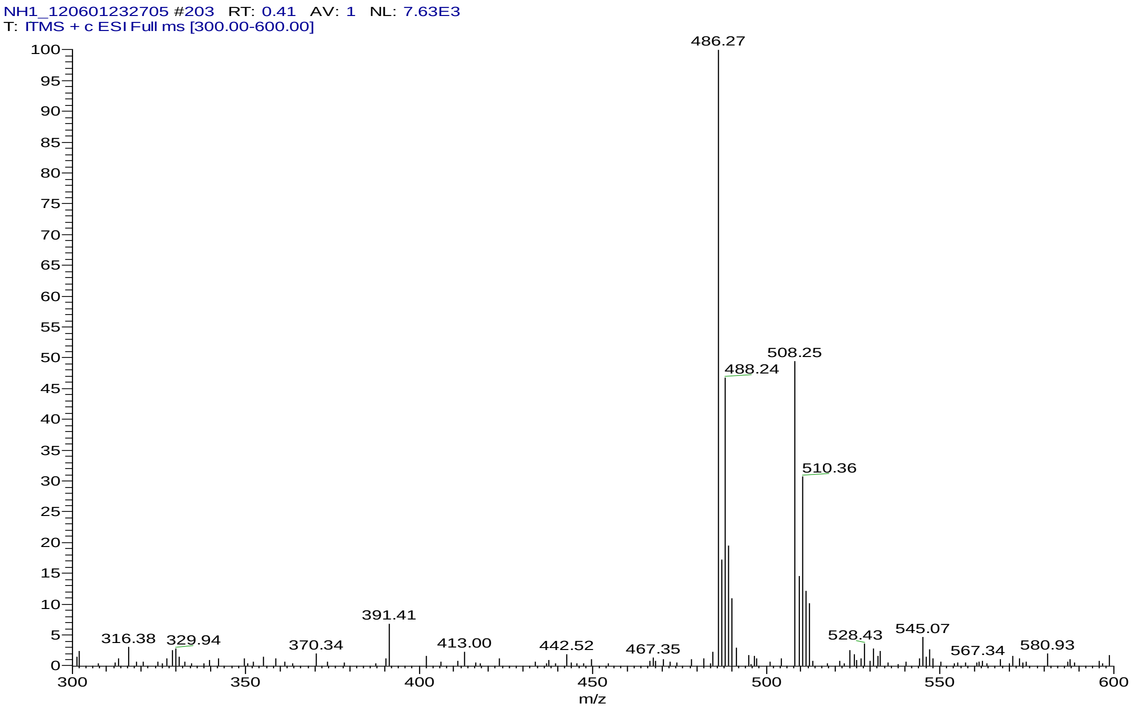

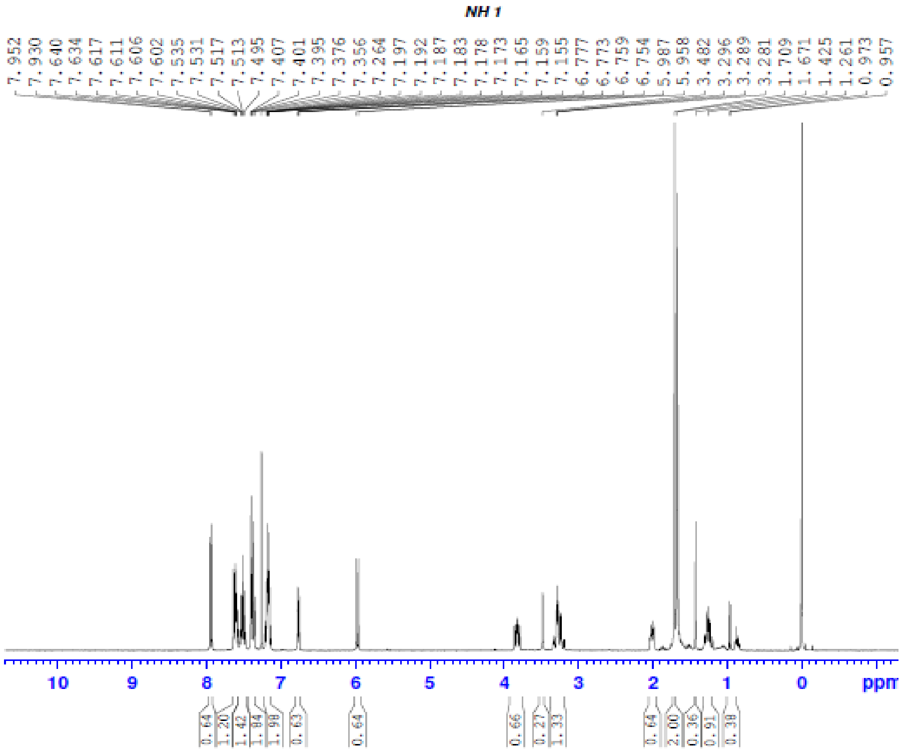

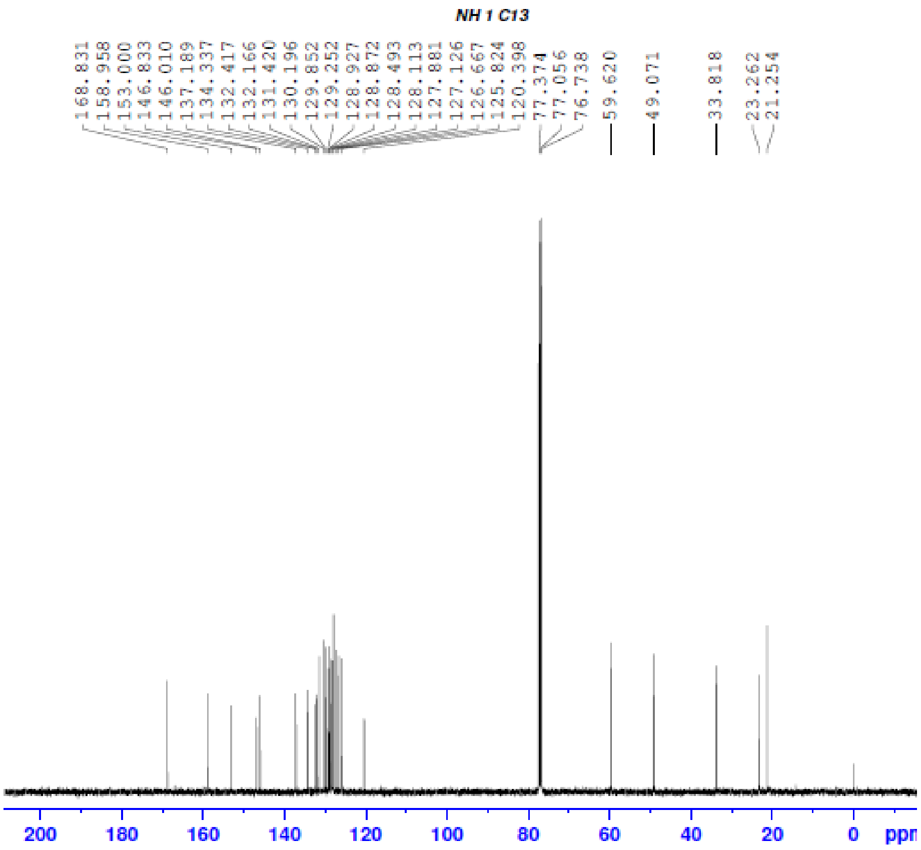

1-(9-chloro-3-(4-chlorophenyl)-11-phenyl-3,3*a*,4,5-tetrahydro-2*H*-pyrazolo[3,4-*a*]acridin-2-yl)ethanone, 4c

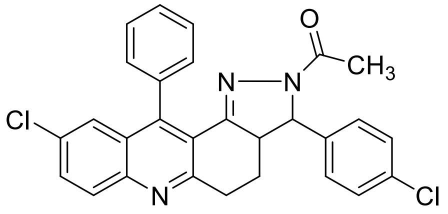

White crystal; M.F: $C_{28}H_{21}Cl_2N_3O$; Yield 35 %; M.P: 192-194 ^{0}C; FT-IR (KBr pellet) ν_{max}/(Cm^{-1}): 1670 (-C=O of tertiary amide); ^{1}H NMR (400 MHz, $CDCl_3$) δ (ppm), 1.74 (s, 3H, $-CH_3$), 2.04-2.16 (m, 1H, $-CH_2$), 2.40-2.46 (m, 1H, $-CH_2$) 3.22-3.30 (m, 1H, $-CH_2$), 3.40-3.42 (dd, *J* = 1.6 Hz, J = 2 Hz, 1H, $-CH_2$), 3.45-3.46 (dd, *J* = 2 Hz, *J* = 2 Hz, 1H, prochiral -CH), 4.86-4.88 (d, *J* = 8 Hz, 1H, chiral -N-CH), 7.09-7.12 (m, 2H), 7.18-7.20 (m, 1H), 7.28-7.31 (m, 1H), 7.35-7.37 (d, *J* = 8.4 Hz, 1H), 7.44-7.45 (d, *J* = 2.4 Hz, 1H), 7.48-7.57 (m, 3H), 7.62-7.63 (d, *J* = 2.4 Hz, 1H), 7.64-7.65 (d, *J* = 2.4 Hz, 1H), 7.95-7.97 (d, *J* = 8.8 Hz,1H); ^{13}C NMR (400 MHz, $CDCl_3$) δ (ppm), 21.57, 27.38, 33.79, 56.30, 65.96, 120.54, 125.82, 127.02, 127.75, 2x127.82, 128.20, 128.26, 128.81, 129.12, 2x129.81, 130.27, 131.44, 132.54, 133.43, 136.67, 139.74, 146.08, 146.49, 152.32, 158.76, 170.12; Exact Mass: 485.11; Found ESI-MS m/z: 486.27 [M+1].

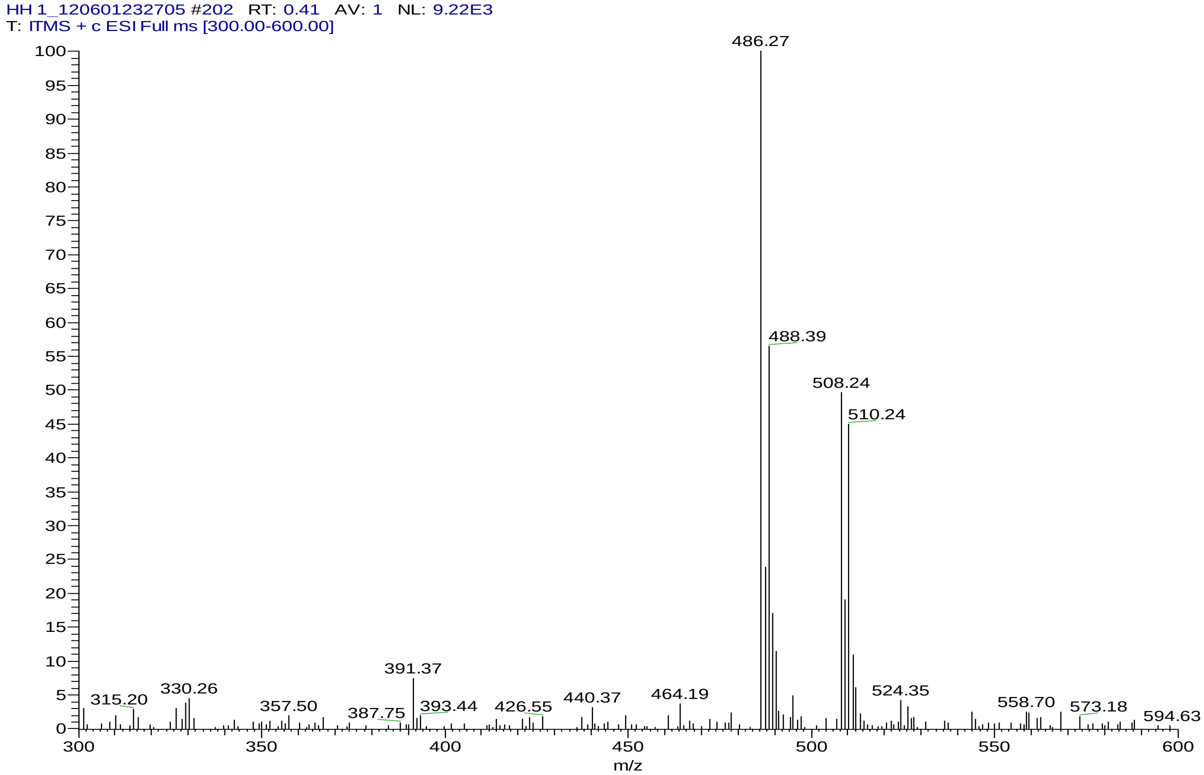
HH 1_120601232705 #202 RT: 0.41 AV: 1 NL: 9.22E3
T: ITMS + c ESI Full ms [300.00-600.00]
486.27
488.39
508.24
510.24
524.35
391.37
315.20
330.26
357.50
387.75
393.44
426.55
440.37
464.19
558.70
573.18
594.63
m/z

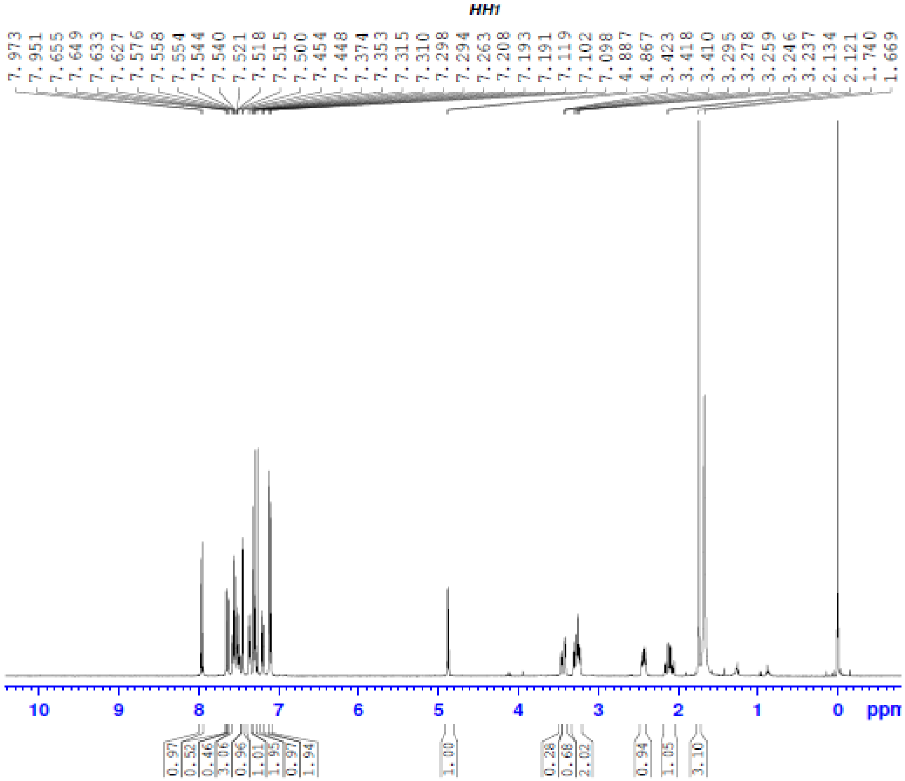
HH1
ppm

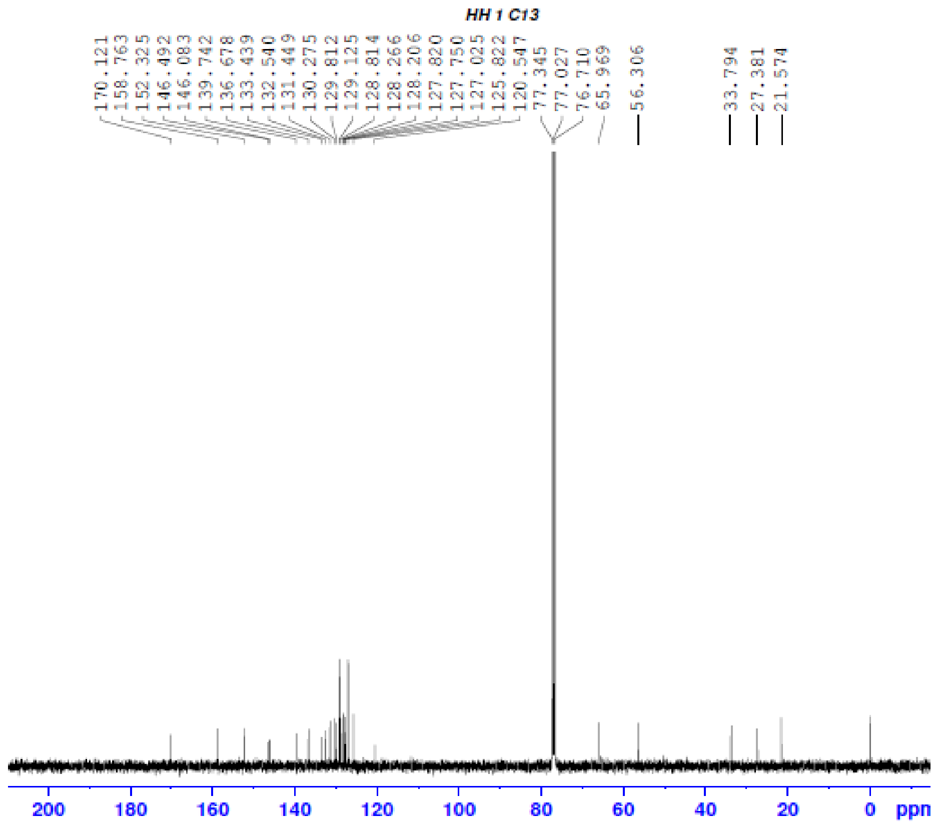

1-(9-chloro-3-(3-methoxyphenyl)-11-phenyl3,3*a*,4,5-tetrahydro-2*H*-pyrazolo[3,4-*a*]acridin-2-yl)ethanone, 4d

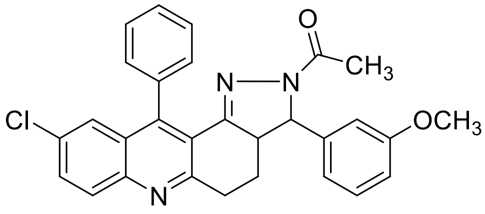

White powder; M.F: $C_{29}H_{24}ClN_3O_2$; Yield 36 %; M.P: 218-220 ^{0}C; FT-IR (KBr pellet) ν_{max}/(Cm^{-1}): 1666 (-C=O of tertiary amide); ^{1}H NMR (400 MHz, $CDCl_3$) δ (ppm), 1.75 (s, 3H, $-CH_3$), 2.04-2.16 (m, 1H, $-CH_2$), 2.41-2.46 (m, 1H, $-CH_2$) 3.20-3.31 (m, 2H, $-CH_2$), 3.39-3.40 (dd, *J* = 1.6 Hz, 1.6 Hz, 1H, prochiral -CH), 3.78 (s, 3H, $-OCH_3$), 4.87-4.89 (d, *J* = 7.6 Hz, 1H, chiral -N-CH), 6.68-6.69 (s, 1H), 6.74-6.80 (m, 2H), 7.16-7.29 (m, 1H), 7.36-7.41 (d, *J* = 8 Hz, 1H), 7.45-7.46 (d, *J* = 7.2 Hz, 1H), 7.47-7.57 (m, 4H), 7.60-7.62 (dd, *J* = 2 Hz, J = 2.4 Hz, 1H), 7.93-7.95 (d, *J* = 8.8 Hz, 1H); ^{13}C NMR (400 MHz, $CDCl_3$) δ: 21.57, 27.52, 33.77, 56.17, 56.36, 66.29, 111.46, 112.52, 117.66, 120.77, 125.75, 127.68, 127.87, 128.14, 128.19, 128.78, 129.86, 130.04, 130.24, 131.29, 132.42, 136.64, 142.97, 145.99, 146.29, 152.36, 158.87, 159.97, 169.93; Exact Mass: 481.16; Found ESI-MS m/z: 482.26 [M+1].

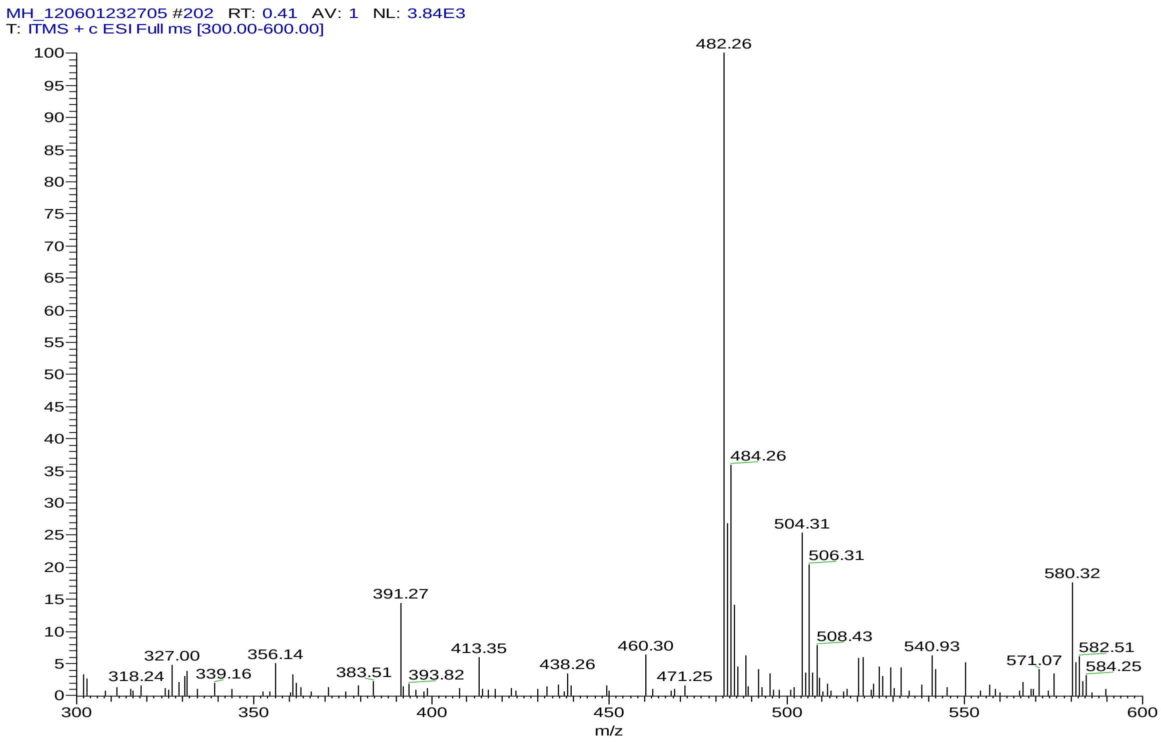
MH_120601232705 #202 RT: 0.41 AV: 1 NL: 3.84E3
T: ITMS + c ESI Full ms [300.00-600.00]
482.26
484.26
504.31
506.31
580.32
391.27
508.43
540.93
582.51
584.25
571.07
413.35
460.30
356.14
327.00
438.26
471.25
383.51
393.82
339.16
318.24
m/z

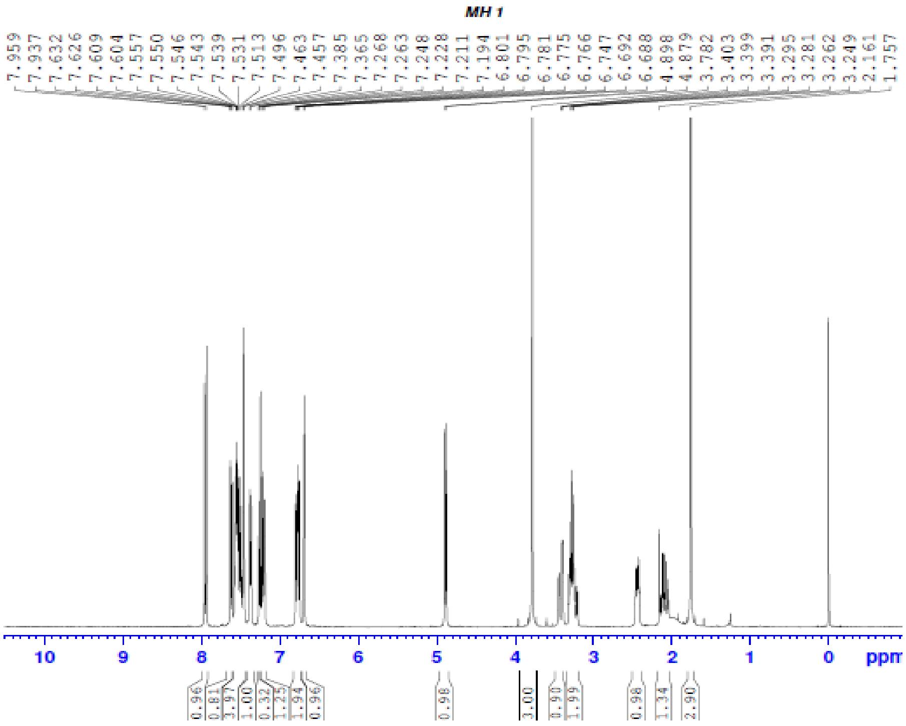
MH 1
7.959 7.937 7.632 7.626 7.609 7.604 7.557 7.550 7.546 7.543 7.539 7.531 7.513 7.496 7.463 7.457 7.385 7.365 7.268 7.263 7.248 7.228 7.211 7.194 6.801 6.795 6.781 6.775 6.766 6.747 6.692 6.688 4.898 4.879 3.782 3.403 3.399 3.391 3.295 3.281 3.262 3.249 2.161 1.757
ppm
0.96 0.81 3.97 1.00 0.32 1.25 1.94 0.96 0.98 3.00 0.90 1.99 0.98 1.34 2.90

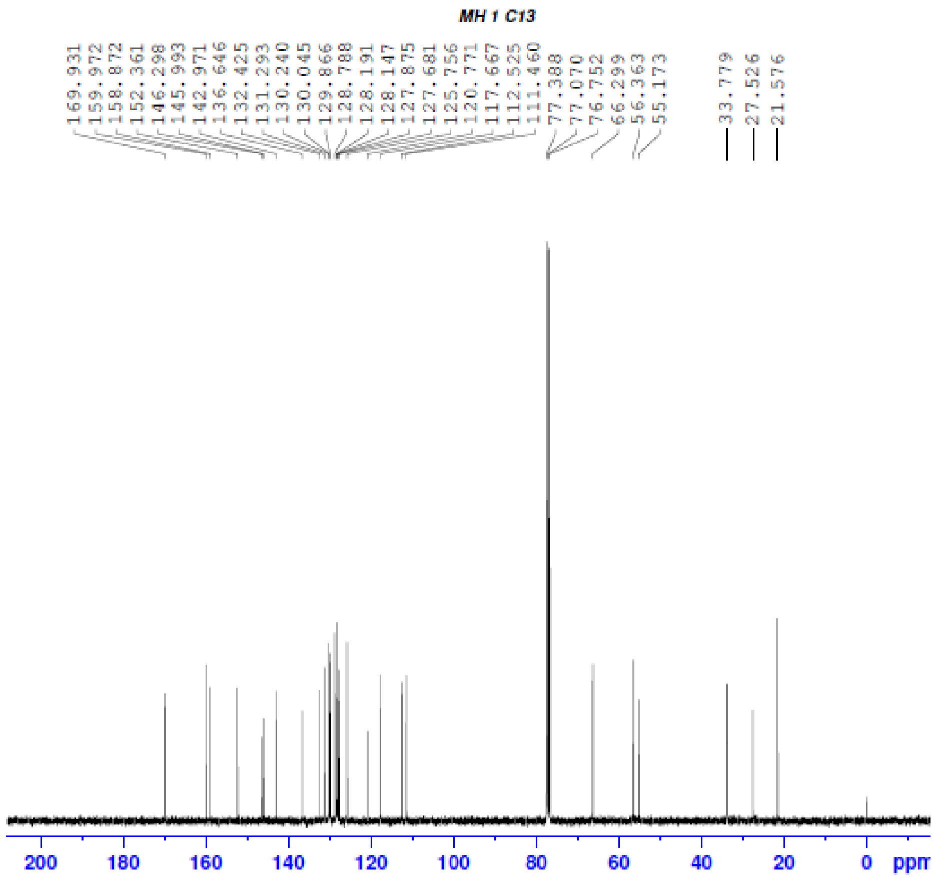

1-(9-chloro-3-(3,4-dimethoxyphenyl)-11-phenyl-3,3a,4,5-tetrahydro-2*H*-pyrazolo[3,4-*a*]acridin-2-yl)ethanone, 4e

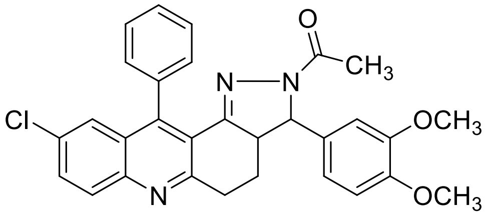

Yellow powder; M.F: $C_{30}H_{26}ClN_3O_3$; Yield 38 %; M.P: 230-232 ^{0}C; FT-IR (KBr pellet) ν_{max}/(Cm^{-1}): 1664 (-C=O of tertiary amide); ^{1}H NMR (400 MHz, $CDCl_3$) δ (ppm), 1.78 (s, 3H, $-CH_3$), 2.06-2.16 (m, 1H, $-CH_2$), 2.44-2.48 (m, 1H, $-CH_2$) 3.26-3.33 (m, 2H, $-CH_2$), 3.43-3.44 (dd, *J* = 1.2 Hz, *J* = 1.2 Hz, 1H, prochiral -CH), 3.86 (s, 3H, $-OCH_3$), 3.88 (s, 3H, $-OCH_3$), 4.88-4.90 (d, *J* = 6 Hz, 1H, chiral -N-CH), 6.63-6.67 (d, *J* = 1.6 Hz, 1H), 6.74-6.76 (dd, *J* = 1.6 Hz, *J* = 1.6 Hz, 1H), 6.83-6.85 (d, *J* = 6.8 Hz, 1H), 7.22-7.23 (m, 1H), 7.41-7.42 (d, *J* = 6 Hz, 1H), 7.48 (d, *J* = 2 Hz, 1H), 7.51-7.53 (m, 2H), 7.56-7.60 (m, 1H), 7.64-7.67 (dd, *J* = 1.6 Hz, *J* = 1.6 Hz, 1H), 7.97-7.99 (d, *J* = 7.2 Hz, 1H); ^{13}C NMR (400 MHz, $CDCl_3$) δ (ppm): 21.65, 27.63, 33.82, 55.86, 56.37, 61.17, 108.81, 111.56, 117.76, 120.93,

125.77, 127.71, 127.81, 128.10, 128.18, 128.23, 128.63, 130.09, 130.30, 131.33, 132.49, 134.01, 136.69, 146.07, 146.21, 148.55, 149.35, 152.52, 158.92, 170.00; Exact Mass: 511.17; Found ESI-MS m/z: 512.27 [M+1].

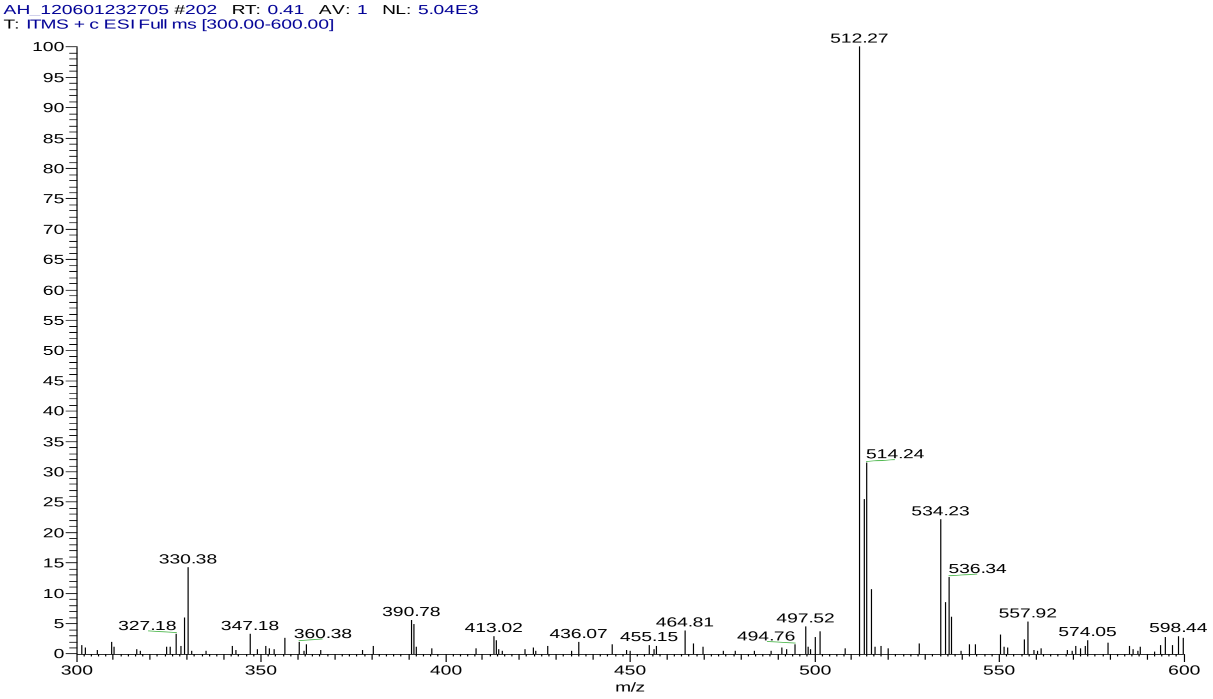

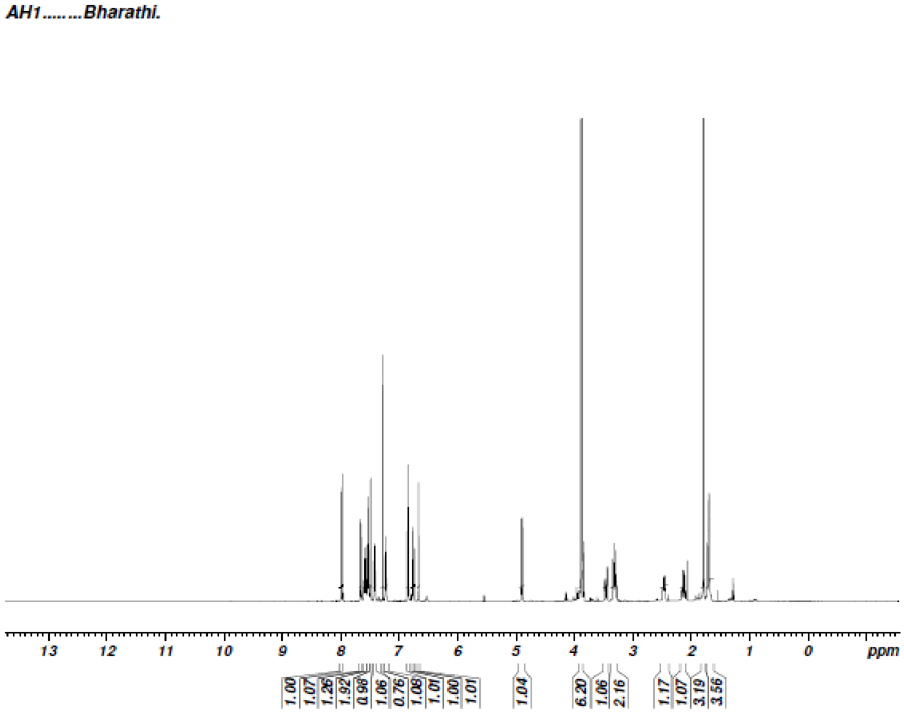

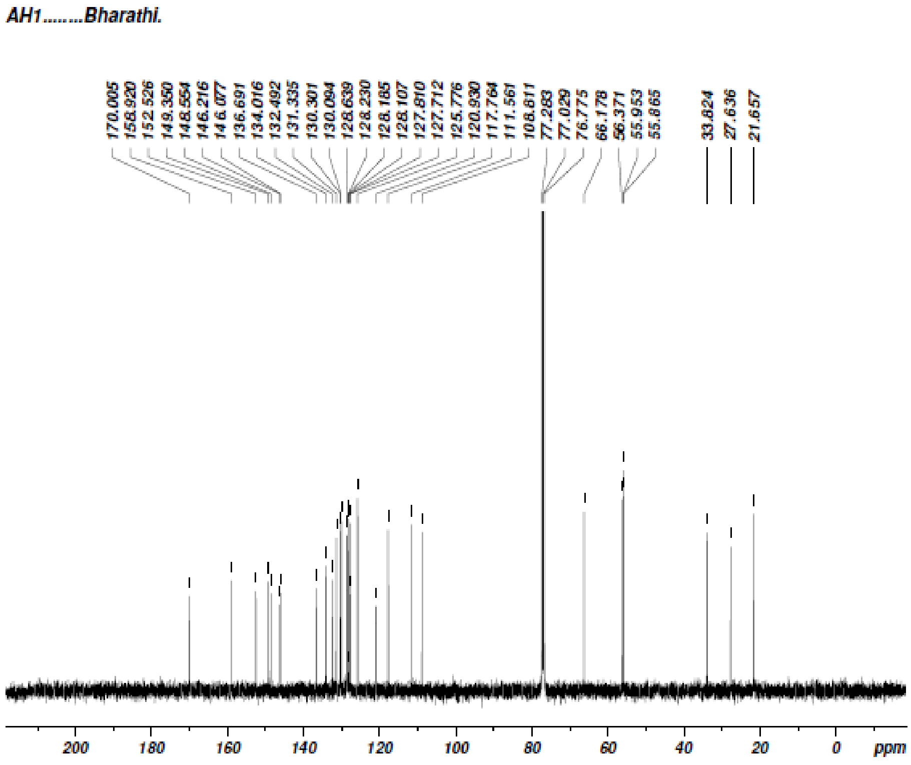

1-(9-chloro-3,11-diphenyl-3*a*,4,5,11*b*-tetrah-ydro-1*H*-pyrazolo[3,4-*a*]acridin-1-yl)ethanone, **5a**

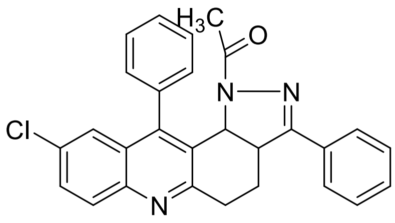

White powder; M.F: $C_{28}H_{22}Cl_2N_3O$; Yield 45 %; M.P: 165-167 ^{0}C; FT-IR (KBr pellet) $\nu_{max}/(Cm^{-1})$: 1658 (-C=O of tertiary amide); ^{1}H NMR (400 MHz, $CDCl_3$) δ (ppm): 1.70 (s, <u>3H</u>, $-CH_3$), 1.81-1.85 (d, *J* = 13.2 Hz, <u>1H</u>, $-CH_2$), 2.06 (s, <u>1H</u>, -CH_2) 3.15-3.24 (m, <u>2H</u>, $-CH_2$), 3.67-3.74 (dt, <u>1H</u>, prochiral -CH), 5.56-5.59 (d, *J* = 11.2 Hz, <u>1H</u>, chiral -N-CH), 6.69-6.98 (d, *J* = 6.8 Hz, <u>2H</u>), 7.17-7.18 (d, *J* = 6.8 Hz, <u>1H</u>), 7.22-7.29 (m, <u>4H</u>), 7.34-7.36 (d, *J* = 7.2 Hz, <u>1H</u>), 7.49-7.53 (m, <u>2H</u>), 7.58-7.62 (t, *J* = 8.2 Hz, <u>2H</u>), 7.93-7.95 (d, *J* = 8.8 Hz, <u>1H</u>); ^{13}C NMR (400 MHz, $CDCl_3$) δ (ppm): 21.34, 23.38, 34.06, 49.60, 62.70, 120.52, 125.98, 126.22, 2x127.93, 128.03, 128.15, 128.23, 128.73, 128.97, 3x129.15, 130.23, 131.55, 132.56, 136.57, 137.46, 146.04, 147.06, 152.87, 159.10, 169.15; Exact Mass: 451.15; Found ESI-MS m/z: 452.30 [M+1].

1-(9-chloro-3-(2-chlorophenyl)-11-phenyl-3*a*,4,5,11*b*-tetrahydro-1*H*-pyrazolo[3,4-*a*]acridin-1-yl)ethanone, 5b

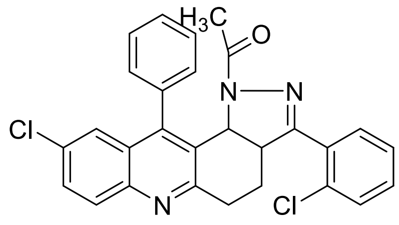

White powder; M.F: $C_{28}H_{21}Cl_2N_3O$; Yield 37 %; M.P: 216-218 ^{0}C; FT-IR (KBr pellet) ν_{max}/(Cm^{-1}): 1676 (-C=O of tertiary amide); ^{1}H NMR (400 MHz, $CDCl_3$) δ (ppm) 1.19-1.30 (m, 1H, -CH_2), 1.70 (s, 3H, -CH_3), 1.97-2.01 (s, 1H, -CH_2), 3.19-3.34 (m, 2H, -CH_2), 3.78-3.86 (m, 1H, prochiral -CH), 5.95-5.98 (d, *J* = 11.6 Hz, 1H, chiral -N-CH), 6.75-6.77 (d, *J* = 7.2 Hz, 1H), 7.15-7.21 (m, 3H), 7.35-7.40 (m, 3H), 7.47-7.55 (m, 2H), 7.58-7.63 (m, 2H), 7.93-7.95 (d, *J* = 8.8 Hz, 1H); ^{13}C NMR (400 MHz, $CDCl_3$, 400 MHz) δ (ppm), 21.27, 23.29, 26.91, 33.85, 49.09, 59.63, 120.41, 125.85, 126.68, 127.13, 127.89, 128.12, 128.50, 128.88, 128.93, 129.27, 129.87, 130.22, 131.44, 132.19, 132.44, 134.36, 137.21, 146.04, 146.84, 152.99, 158.97, 168.83; Exact Mass: 485.11; Found ESI-MS m/z: 486.27 [M+1].

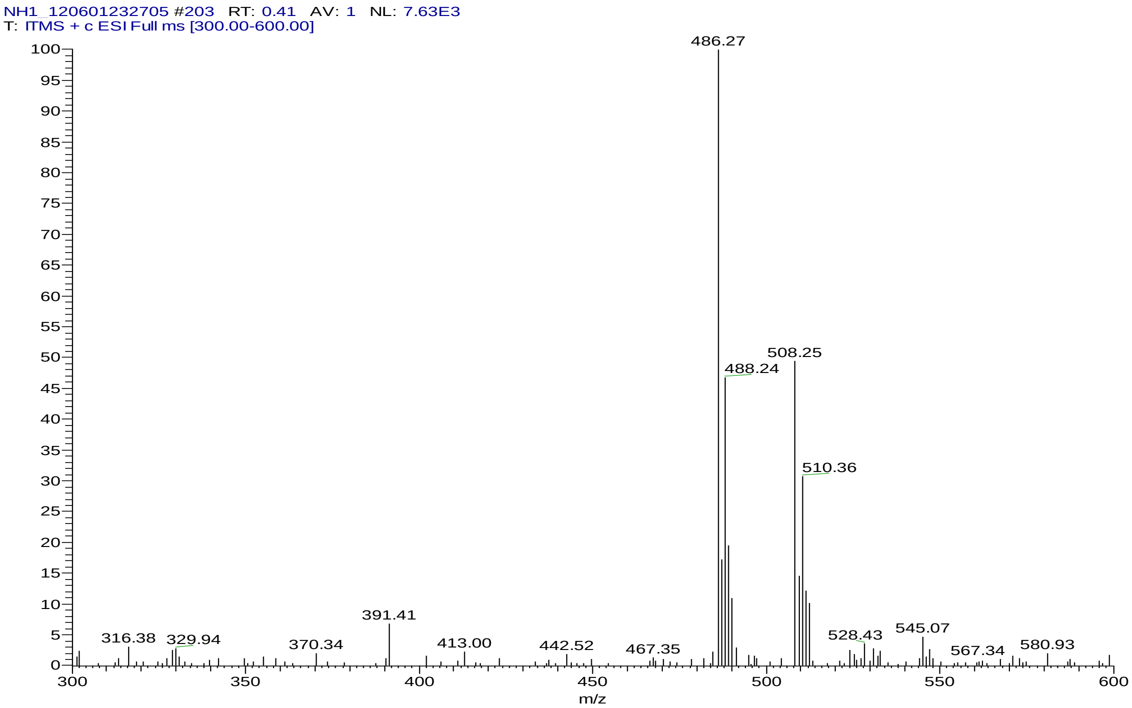

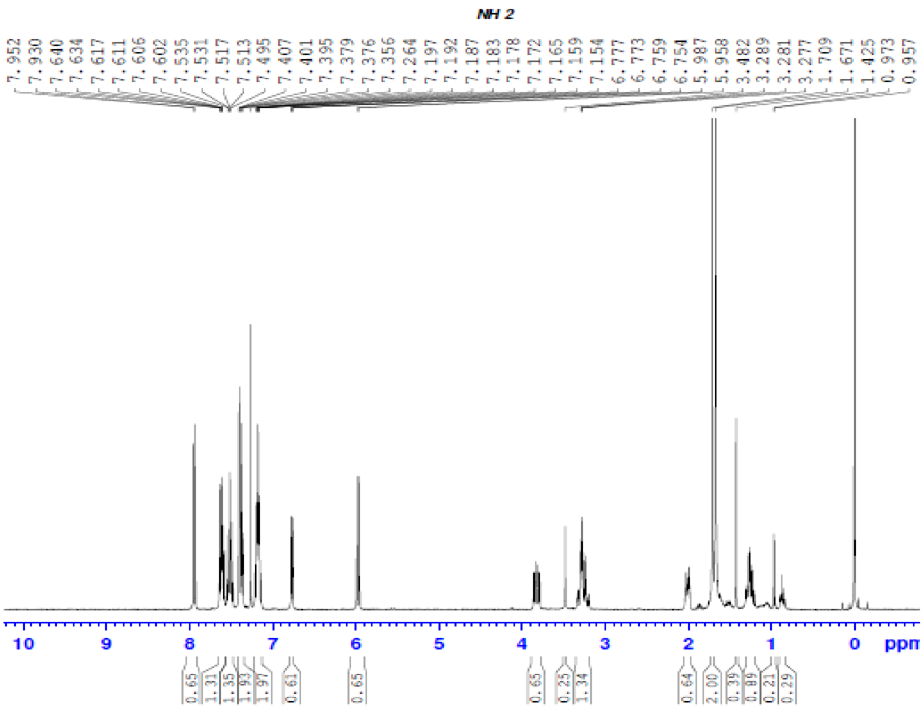
NH 2
7.952
7.930
7.640
7.634
7.617
7.611
7.606
7.602
7.535
7.531
7.517
7.513
7.495
7.407
7.401
7.395
7.379
7.376
7.356
7.264
7.197
7.192
7.187
7.183
7.178
7.172
7.165
7.159
7.154
6.777
6.773
6.759
6.754
5.987
5.958
3.482
3.289
3.281
3.277
1.709
1.671
1.425
0.973
0.957
10
9
8
7
6
5
4
3
2
1
0
ppm
0.65
1.31
1.35
1.93
1.97
0.61
0.65
0.65
0.25
1.34
0.64
2.00
0.39
0.89
0.21
0.29

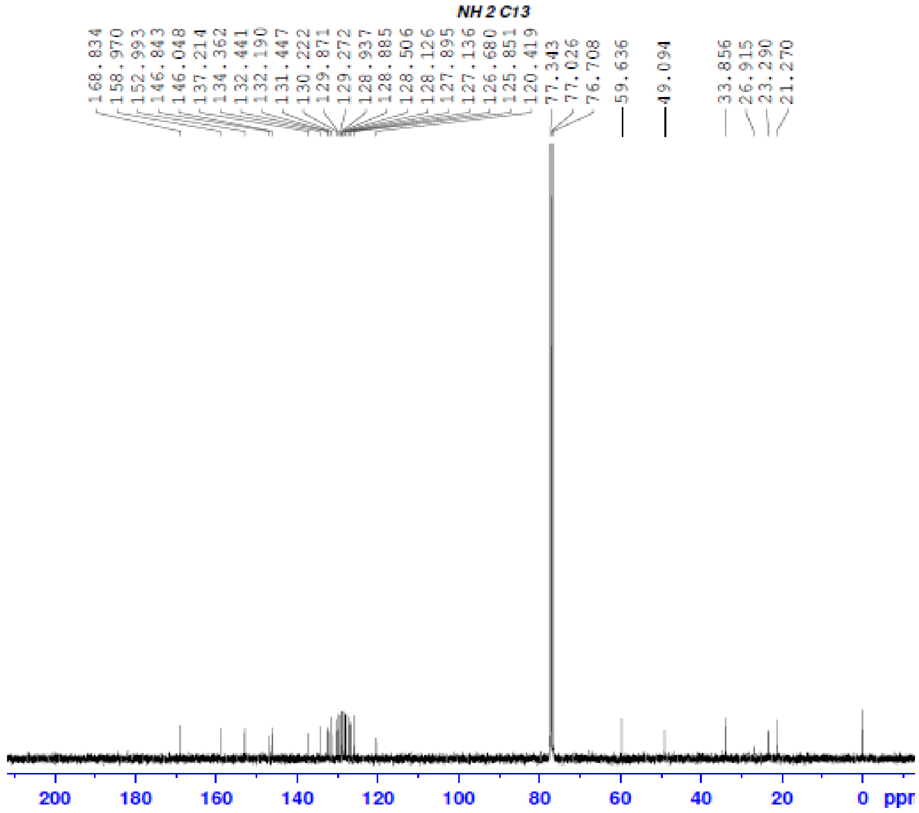
NH 2 C13
168.834
158.970
152.993
146.843
146.048
137.214
134.362
132.441
132.190
131.447
130.222
129.871
129.272
128.937
128.885
128.506
128.126
127.895
127.136
126.680
125.851
120.419
77.343
77.026
76.708
59.636
49.094
33.856
26.915
23.290
21.270
200
180
160
140
120
100
80
60
40
20
0
ppm

1-(9-chloro-3-(4-chlorophenyl)-11-phenyl-3*a*,4,5,11*b*-tetrahydro-1*H*-pyrazolo[3,4-*a*]acridin-1-yl)ethanone, 5c

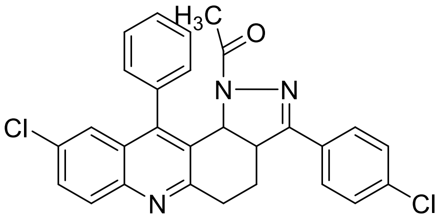

Brown solid; M.F: $C_{28}H_{21}Cl_2N_3O$; Yield 42 %; M.P: 178-180 ^{0}C; FT-IR (KBr pellet) ν_{max}/(Cm^{-1}): 1656 (-C=O of tertiary amide); ^{1}H NMR (400 MHz, $CDCl_3$) δ (ppm), 1.69 (s, 3H, $-CH_3$), 1.81-1.84 (m, 1H, $-CH_2$), 2.04-2.05 (d, *J* = 6.4 Hz, 1H, $-CH_2$), 3.08-3.24 (m, 1H, $-CH_2$), 3.30-3.35 (m, 1H, $-CH_2$), 3.66-3.74 (dt, 1H, prochiral -CH), 5.52-5.55 (d, *J* = 11.2 Hz, 1H, chiral -N-CH), 6.91-6.93 (d, *J* = 8 Hz, 2H), 7.16-7.18 (d, *J* = 7.2 Hz, 1H), 7.24-7.26 (d, *J* = 6.9, 2H), 7.33-7.35 (d, *J* = 7.6 Hz, 2H), 7.39 (s, 1H), 7.45-7.54 (m, 1H), 7.58-7.63 (m, 2H), 7.93-7.95 (d, *J* = 9.2 Hz, 1H); ^{13}C NMR (400 MHz, $CDCl_3$) δ (ppm), 21.18, 23.38, 33.86, 49.31, 61.91, 120.19, 125.84, 127.53, 127.86, 2x127.96, 128.13, 128.59, 128.85, 128.90, 2x129.00, 130.14, 131.49, 132.47, 133.59, 135.08, 137.23, 145.97, 146.99, 152.75, 158.77, 168.97; Exact Mass: 485.11; Found ESI-MS m/z: 486.27 [M+1].

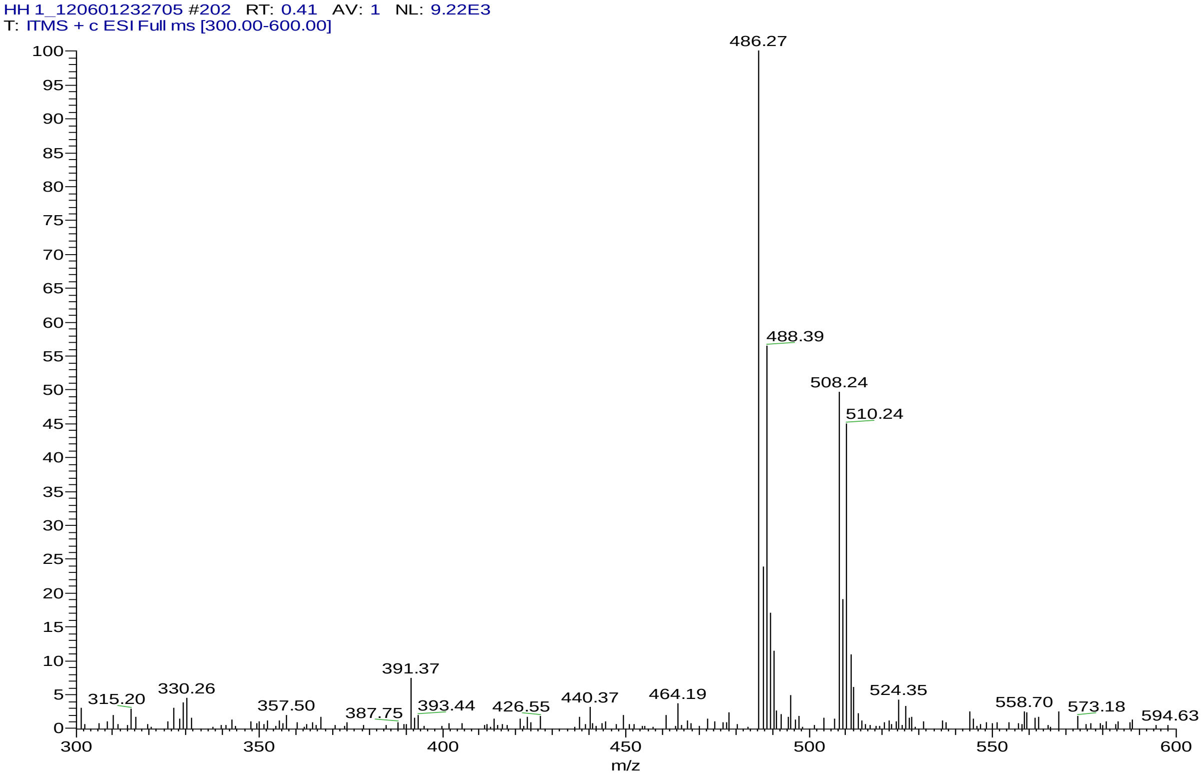

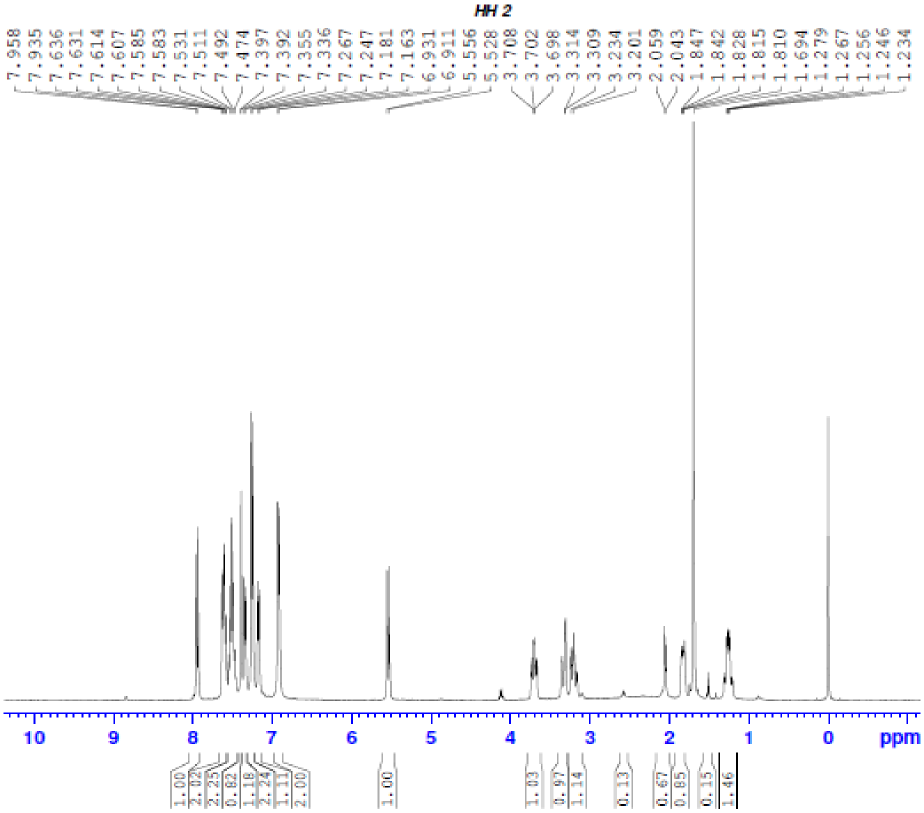
HH 2
ppm

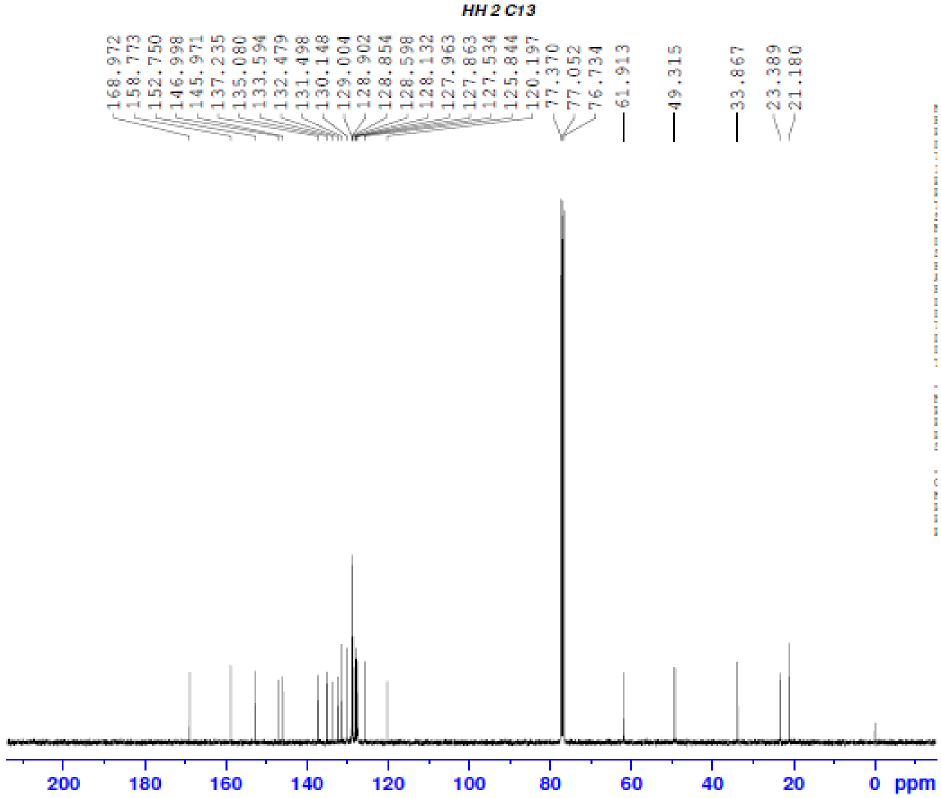
HH 2 C13
ppm

1-(9-chloro-3-(3-methoxyphenyl)-11-phenyl-3*a*,4,5,11*b*-tetrahydro-1*H*-pyrazolo[3,4-*a*]acridin1-yl)ethanone, 5d

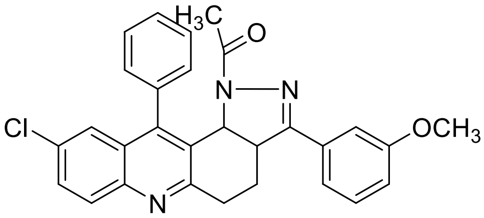

White powder; M.F: $C_{29}H_{24}ClN_3O_2$; Yield 39 %; M.P: 140-142 ^{0}C; FT-IR (KBr pellet) ν_{max}/(Cm^{-1}): 1666 (-C=O of tertiary amide); ^{1}H NMR (400 MHz, $CDCl_3$) δ (ppm), 1.23-1.34 (dd, *J* = 1.6 Hz, 1.6 Hz, 1H, prochiral -CH), 1.64 (s, 3H, $-CH_3$), 1.86-1.89 (m, 2H, $-CH_2$), 3.15-3.34 (m, 2H, $-CH_2$), 3.71 (s, 3H, $-OCH_3$), 5.52-5.55 (d, *J* = 11.2 Hz, 1H, chiral -N-CH), 6.51 (s, 1H), 6.56-6.57 (d, *J* = 7.2 Hz, 1H), 6.75-6.77 (dd, *J* = 2 Hz, *J* = 1.6 Hz, 1H), 7.16-7.20 (m, 2H), 7.33-7.39 (m, 2H), 7.47-7.55 (m, 2H), 7.60-7.63 (m, 2H), 7.92-7.94 (d, *J* = 8.8 Hz, 1H); ^{13}C NMR (400 MHz, $CDCl_3$) δ: 21.21, 23.08, 34.01, 49.45, 55.12, 62.46, 112.37, 112.51, 118.46, 120.35, 125.84, 127.89, 127.99, 128.07, 128.60, 128.81, 129.02, 129.76, 130.18, 131.37, 132.38, 137.37, 138.09, 145.96, 146.83, 152.70, 158.98, 159.78, 168.97; Exact Mass: 481.16; Found ESI-MS m/z: 482.56 [M+1].

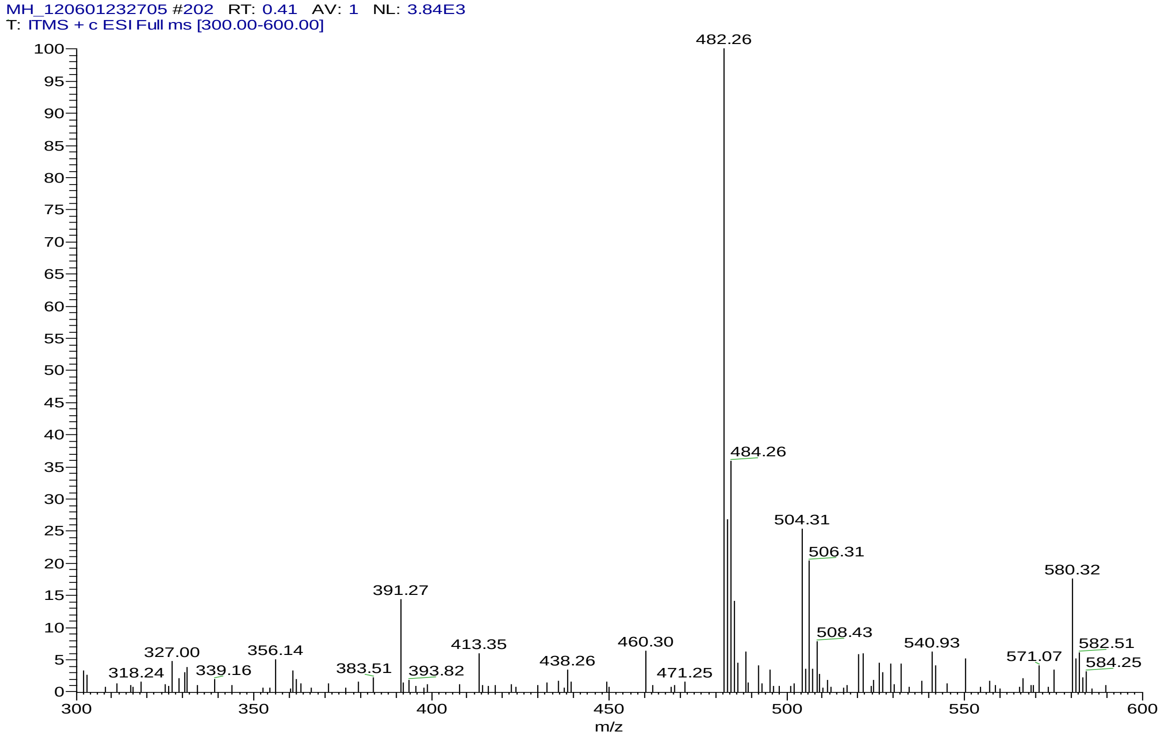

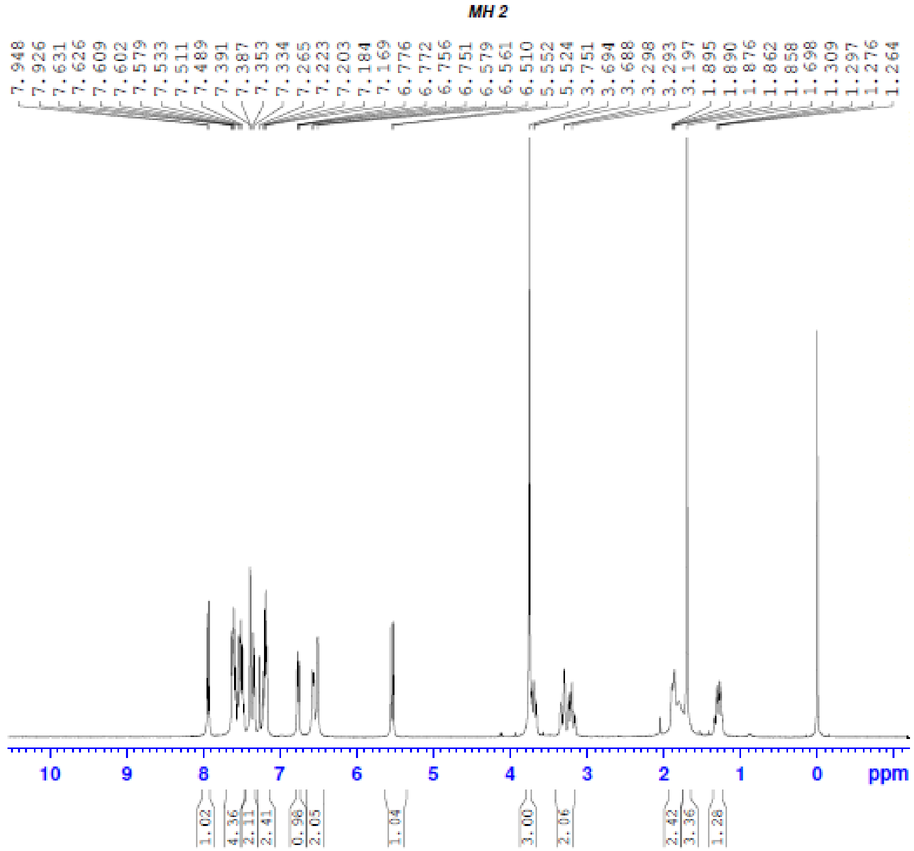
MH 2
7.948
7.926
7.631
7.626
7.609
7.602
7.579
7.533
7.511
7.489
7.391
7.387
7.353
7.334
7.265
7.223
7.203
7.184
7.169
6.776
6.772
6.756
6.751
6.579
6.561
6.510
5.552
5.524
3.751
3.694
3.688
3.298
3.293
3.197
1.895
1.890
1.876
1.862
1.858
1.698
1.309
1.297
1.276
1.264
10
9
8
7
6
5
4
3
2
1
0
ppm
1.02
4.36
2.11
2.41
0.98
2.05
1.04
3.00
2.06
2.42
3.36
1.28

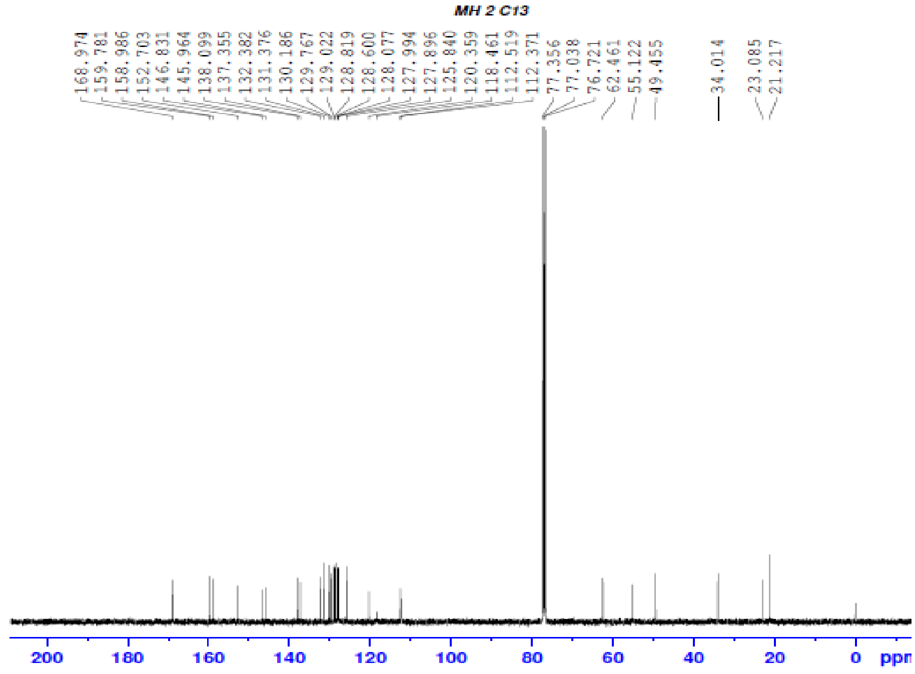
MH 2 C13
168.974
159.781
158.986
152.703
146.831
145.964
138.099
137.355
132.382
131.376
130.186
129.767
129.022
128.819
128.600
128.077
127.994
127.896
125.840
120.359
118.461
112.519
112.371
77.356
77.038
76.721
62.461
55.122
49.455
34.014
23.085
21.217
200
180
160
140
120
100
80
60
40
20
0
ppm

1-(9-chloro-3-(3,4-dimethoxyphenyl)-11-phenyl-3a,4,5,11b-tetrahydro-1*H*-pyrazolo[3,4-*a*]acridin-1-yl)ethanone, 5e

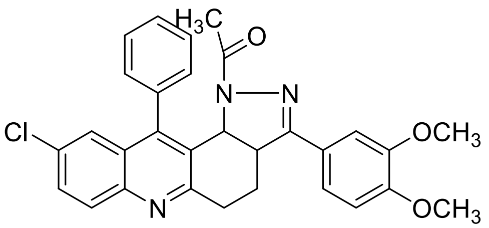

White powder; M.F: $C_{30}H_{26}ClN_3O_3$; Yield 39 %; M.P: 236-238 ^{0}C; FT-IR (KBr pellet) ν_{max}/(Cm^{-1}): 1654 (-C=O of tertiary amide); ^{1}H NMR (400 MHz, $CDCl_3$) δ (ppm), 1.26-1.37 (m, 1H, $-CH_2$), 1.72 (s, 3H, $-CH_3$), 1.87-1.92 (m, 1H, $-CH_2$), 3.19-3.26 (m, 1H, $-CH_2$), 3.31-3.37 (m, 1H, $-CH_2$), 3.66-3.72 (m, 1H, prochiral -CH), 3.83 (s, 3H, $-OCH_3$), 3.84 (s, 3H, $-OCH_3$), 5.53-5.55 (d, *J* = 8.8 Hz, 1H, chiral -N-CH), 6.52 (s, 2H), 6.78-6.79 (d, *J* = 6.4 Hz, 1H), 7.20-7.22 (m, 1H), 7.35 (m, 1H), 7.41 (d, *J* = 2 Hz, 1H), 7.49-7.56 (m, 2H), 7.57-7.59 (m, 1H), 7.60-7.65 (m, 1H), 7.95-7.97 (d, *J* = 7.2 Hz, 1H); ^{13}C NMR (400 MHz, $CDCl_3$) δ: 21.24, 23.08, 34.06, 55.84, 55.87, 62.41, 109.55, 111.36, 118.14, 120.39, 125.83, 2x128.02, 128.10, 128.56, 128.87, 128.89, 2x129.10, 130.23, 131.39, 132.42, 137.41, 146.00, 146.79, 148.59, 149.11, 152.78, 159.04, 169.99; Exact Mass: 511.17; Found ESI-MS m/z: 512.27 [M+1].

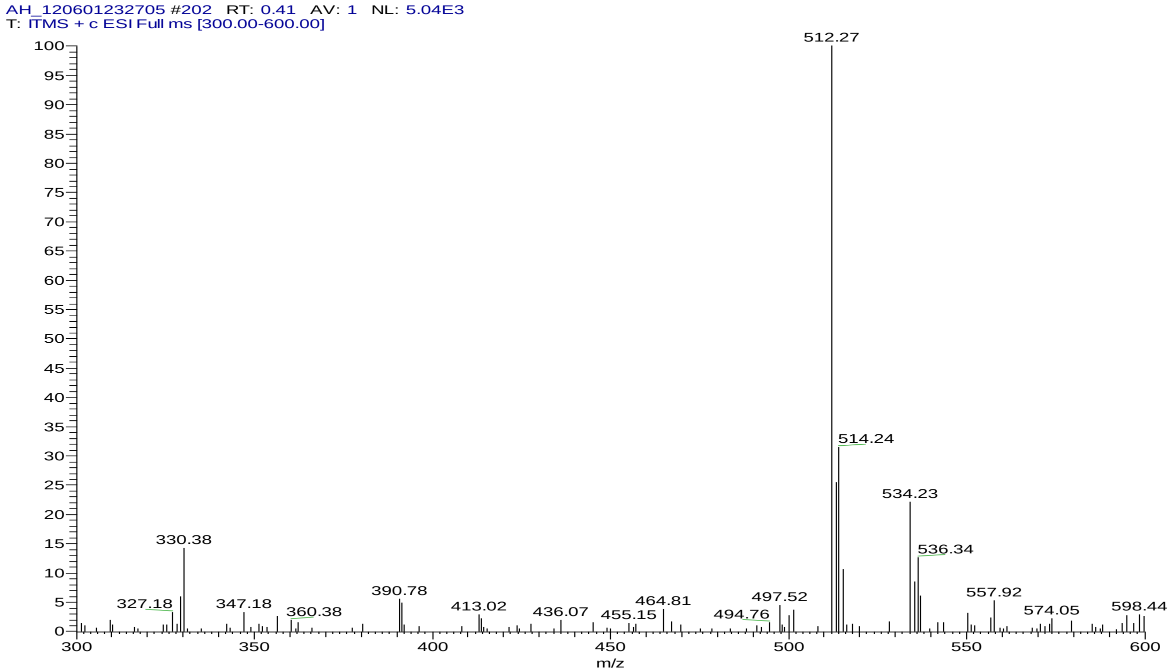

AH2........Bharathi.

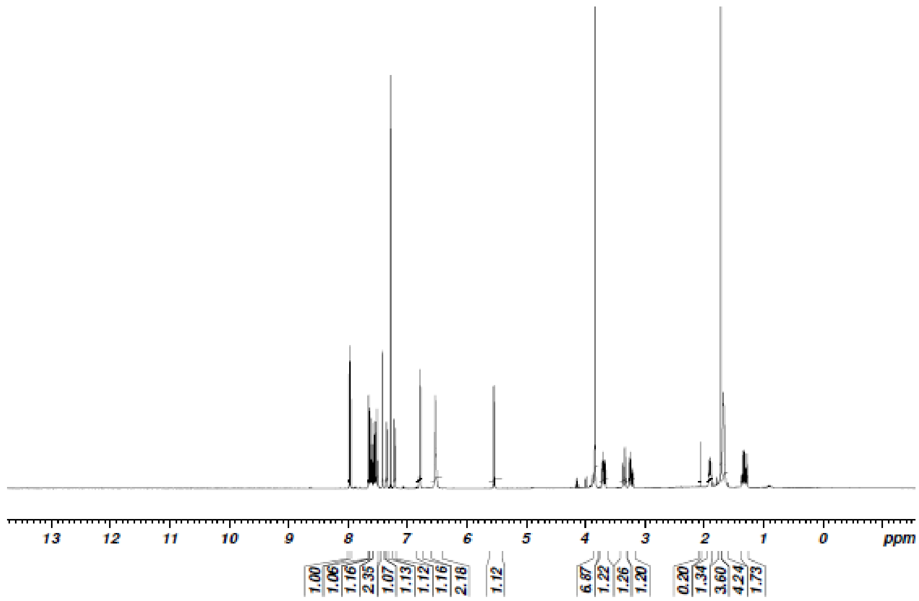

AH2........Bharathi.

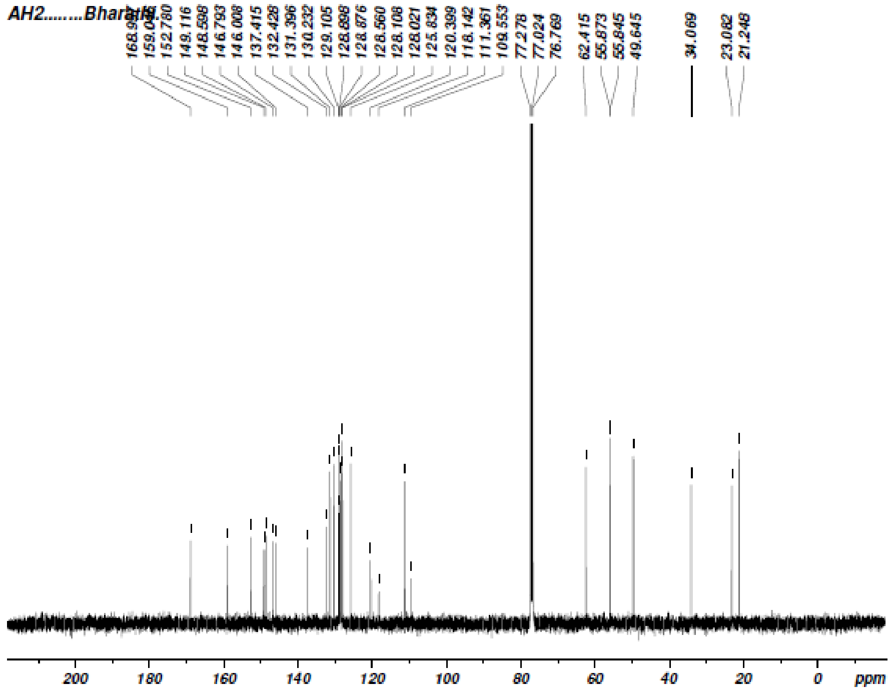

Supplement: Supplementary Information [file srep39753-s1.pdf]
